# Xyloglucan endotransglycosylase/hydrolase increases tightly-bound xyloglucan and chain number but decreases chain length contributing to the defense response that *Glycine max* has to *Heterodera glycines*

**DOI:** 10.1371/journal.pone.0244305

**Published:** 2021-01-14

**Authors:** Prakash M. Niraula, Xuefeng Zhang, Dragica Jeremic, Katherine S. Lawrence, Vincent P. Klink

**Affiliations:** 1 Department of Biological Sciences, Mississippi State University, Starkville, Mississippi State, United States of America; 2 Department of Sustainable Bioproducts, Mississippi State University, Starkville, Mississippi State, United States of America; 3 Department of Entomology and Plant Pathology, Auburn University, Auburn, Alabama, United States of America; 4 Department of Biochemistry, Molecular Biology, Entomology and Plant Pathology, Mississippi State University, Starkville, Mississippi State, United States of America; 5 Center for Computational Sciences High Performance Computing Collaboratory, Starkville, Mississippi State, United States of America; University of California Riverside, UNITED STATES

## Abstract

The *Glycine max* xyloglucan endotransglycosylase/hydrolase (EC 2.4.1.207), GmXTH43, has been identified through RNA sequencing of RNA isolated through laser microdissection of *Heterodera glycines*-parasitized root cells (syncytia) undergoing the process of defense. Experiments reveal that genetically increasing XTH43 transcript abundance in the *H*. *glycines*-susceptible genotype *G*. *max*_[Williams 82/PI 518671]_ decreases parasitism. Experiments presented here show decreasing XTH43 transcript abundance through RNA interference (RNAi) in the *H*. *glycines*-resistant *G*. *max*_[Peking/PI 548402]_ increases susceptibility, but it is unclear what role XTH43 performs. The experiments presented here show XTH43 overexpression decreases the relative length of xyloglucan (XyG) chains, however, there is an increase in the amount of those shorter chains. In contrast, XTH43 RNAi increases XyG chain length. The experiments show that XTH43 has the capability to function, when increased in its expression, to limit XyG chain extension. This outcome would likely impair the ability of the cell wall to expand. Consequently, XTH43 could provide an enzymatically-driven capability to the cell that would allow it to limit the ability of parasitic nematodes like *H*. *glycines* to develop a feeding structure that, otherwise, would facilitate parasitism. The experiments presented here provide experimentally-based proof that XTHs can function in ways that could be viewed as being able to limit the expansion of the cell wall.

## Introduction

The plant cell wall (CW) is a dynamic frame surrounding the enclosed protoplast composed of approximately 30% cellulose, 30% hemicellulose, 35% pectin and 1–5% structural proteins. Cellulose and hemicellulose mostly provide rigidity to the wall while pectin provides fluidity through the gelatinous polysaccharide matrix [1]. Cellulose is interlocked by a cross-linked non-cellulosic polysaccharide matrix, the hemicelluloses, having a (1→4)-linked mono-sugar xyloglucan (XyGs), xylan, mannan and glucomannan backbone [2–4].

XyG is the principal hemicellulose, composing up to 25% of the cell wall. XyG is hydrogen-bonded to adjacent cellulose microfibril surfaces, forming a network that may limit CW extensibility by tethering adjacent microfibrils as well as regulating cell enlargement and acting as a load bearing structure [4]. XyG is a branched polysaccharide also having a (β1–4)-linked D-glucan backbone [5]. In addition to the glucan backbone, three out of four glucose residues are substituted with α-xylosyl residues attached to the 6-position of β-glucosyl residues, and the terminal galactose is attached to the 2-position of xylosyl residues by β-linkage [6]. XyG heterogeneity occurs from either differences in molecular mass or the distribution of additional branching of galactosyl or fucosyl residues [7–9]. Two kinds of XyGs exist, with molecular masses of 60 kDa and 180 kDa [10]. Each XyG is constructed of two kinds of oligosaccharide repeating units, a heptasaccharide (glucose/xylose, 4:3) and nonsaccharide (glucose/xylose/galactose/fucose, 4:3:1:1). Fucosylated subunits are predominant in *G*. *max* root XyG, characteristically similar to the bulk of *Arabidopsis thaliana* (thale cress) root XyG [11,12]. The synthesis of the XyG backbone is believed to involve cellulose synthase-like family genes [13]. Since, the exogenous XyG fails to complex with newly formed cellulose microfibrils, the macromolecular organization involves the secretion of polysaccharides [10,14]. Golgi bodies are involved in the synthesis and secretion of non-cellulosic polysaccharides destined for CW incorporation [15]. XyG backbone synthesis is believed to occur in the Golgi cisternae, while further branching of galactosylation and fucosylation may occur in the medial- and trans- Golgi as XyG matures [16,17]. Golgi-synthesized matrix polysaccharides are transported to plasma membrane and apoplast by the conventional pathway via secretory vesicles [18]. The secretory vesicle clusters (SVCs) containing the secretory carrier membrane protein mediate polysaccharide secretion during exocytosis and endocytosis during cell plate formation [19,20]. XyG performs key roles in cell enlargement and XyG biosynthesis and metabolism, remodeling and organization [21,22]. Consequently, XyG has an impact on growth regulation and biotic and abiotic stress responses [21,22]. The cross-linking by XyG between parallel microfibrils may function as a bracket linking the parallel fibrils as a beam [23]. XyG has structural and signaling roles and modifies the physical characteristics of oligosaccharides or polysaccharides through their incorporation. Partial XyG hydrolysis yields apoplastic XyG-derived oligosaccharides (XGOs) that modulate plant growth, morphogenesis and signal transduction [24].

Xyloglucan endotransglycosylase/hydrolase (XTH) (EC 2.4.1.207) is an ancient XyG-metabolizing protein found in all land plants, evidence indicating their presence and activity predates land colonization [7,25–35]. *A*. *thaliana* has 33 XTHs, one-third of them existing in 2–4 gene clusters [36,37]. XTH cuts and rejoins XyG chains that interconnect adjacent microfibrils, restructuring CWs by reversibly or irreversibly loosening them to permit cell expansion [31]. CW restructuring also happens through integration of newly synthesized XyGs [31]. XTHs have a signal peptide, allowing targeting to the ER and can be *N*-glycosylated, indicating secretory pathway processing [35,38–44]. Promoter element analyses link lysigeny and the development of a plant parasitic nematode feeding structure (syncytium) that forms through nematode-mediated activities [45]. XTHs are expressed in tissues infected with pathogens [46]. In *G*. *max*, infection by the plant parasitic nematode *Heterodera glycines* leads to the development of a syncytium created through localized CW degradation caused by enzymatic activities of the nematode. XTH (XTH43) is highly expressed within syncytia undergoing a defense response and overexpression leads to resistance [47,48]. XTH overexpression in *Populus* sp. results in the initial shortening of XyG chain length which is later unaffected by the higher levels of XTH as the cells mature, maintaining a well-defined cell boundary [49]. This work provides mechanistic insight into how XTH limits cellular expansion. However, as already stated, other plausible mechanisms of the relationship of XTH and cell walls may relate to the already-described XGOs [24]. As an important signal transduction platform, a detailed analysis of the entire mitogen activated protein kinase (MAPK) family of *G*. *max* as it relates to its defense to *H*. *glycines* parasitism has shown a number of them function in the process, leading to the expression of different suites of proven defense genes [50]. RNA sequencing has revealed the relative transcript abundances of different suites of genes are regulated by these MAPKs while also a core set of syncytium expressed genes are regulated by these MAPKs [51,52]. Consequently, a number of different routes that lead to the failure of the syncytium to form could exist, but they may or may not all involve XTHs in one way or another. Nishikubo *et al*. [49] stated XyG chain length determines whether the CW matrix is accessible to enzymatic degradation with shorter chain length preventing their access and activity. The cell wall modifications that *G*. *max* employs through XTH43 function could effectively counteract the activities initiated by *H*. *glycines*, directly or indirectly, that would normally lead to the production of a syncytium. By doing so, *G*. *max* appears to have a simple way to prevent cellular expansion and syncytium development that impairs *H*. *glycines*. Alternatively, these cell wall modifying processes mediated by XTH43 occur after *H*. *glycines* has already been overcome by other cellular processes initiated by *G*. *max* and are part of a recovery process involving XTH43 that occurs after an earlier defense process has reached its conclusion. In either event, XTH43 appears to perform an important role [47,48].

This study examines the role of *G*. *max* XTH43. A CW polysaccharide analysis is performed by polysaccharide fractionation proceeding with a XyG assay and gel permeation chromatography (GPC) of both genetically engineered *G*. *max* and control root samples of both genetically engineered *G*. *max* and control root samples. The results provide insight into the mechanism by which XTH43 limits cellular expansion or strengthens the CW through XyG remodeling or/and integration of newly synthesized and secreted XyG during the defense response.

## Materials and methods

### Generation of transgenic plants

The *G*. *max* XTH43 gene (XTH43) has been cloned [48]. The procedures used to generate XTH43-overexpressing (XTH43-OE) and XTH43-RNAi roots and used in the analyses here have been described, but are summarized here [48]. The pRAP15 plasmid has been used for XTH43 overexpression (XTH43-OE) [53]. The pRAP17 plasmid has been used for XTH43-RNAi [54]. XTH43 gene expression in both the pRAP15-OE and pRAP17-RNAi plasmids is driven by the figwort mosaic virus (FMV) sub-genomic transcript (Sgt) promoter [53–55]. The FMV-Sgt promoter consists of a 301 bp FMV Sgt promoter fragment (sequence -270 to +31 from the transcription start site (TSS) [55]. The RNAi cassette of pRAP17 is designed to produce a hairpin RNA having inverted repeats [54,56]. A solution of *Agrobacterium rhizogenes* strain K599 (K599) has been transformed with the appropriate XTH43-OE, pRAP15 (OE control), XTH43-RNAi or pRAP17 (RNAi control) vector. The genetically transformed K599 has been centrifuged to pellet the bacteria. The K599 has been re-suspended in Murashige and Skoog (MS) media, including vitamins (Duchefa Biochemie; The Netherlands) and 3.0% sucrose at a pH of 5.7 (MS media) [54,57]. The roots from 1 week old *H*. *glycines*_[NL1-Rhg/HG-type 7/race 3]_-susceptible *G*. *max*_[Williams 82/PI 518671]_ (for XTH43-OE) or *H*. *glycines*_[NL1-Rhg/HG-type 7/race 3]_-resistant *G*. *max*_[Peking/PI 548402]_ (for XTH43-RNAi) have been excised at the hypocotyl while in a Petri dish containing a slurry of K599 with a new, sterile razor blade that has been used for each individual tested genetic construct. The procedure allows the K599 access to the plant tissue through the wound site. Subsequently, 25 root-less plants have been placed in a 140 ml glass beaker containing 25 ml of transformed K599 in the MS media solution. The root-less plants, housed within the beaker containing the K599, have then been placed under a vacuum for 5 minutes. The plants have been left in the vacuum for 10 minutes, allowing the K599 ample time to gain entry to the plant tissue. The vacuum has then been slowly released, ensuring the transformed K599 remains in the plant tissue while also ensuring air bubbles do not enter the plant tissue. After this co-cultivation period, the cut ends of root-less *G*. *max* plants have been placed individually 3–4 cm deep into fresh coarse vermiculite (Palmetto Vermiculite) in 50-cell flats (T.O. Plastics). The 50-cell flats are placed in 61.9 (L) X 38.4 (W) X 15.6 (H) semi-clear plastic chambers (Sterlite^®^). At this point, the plants have been covered to permit the build-up of humidity which prevents the root-less plants from drying out and time to regenerate roots. The humidity chambers have been placed at a distance of 20 cm from standard fluorescent cool white 4,100 K, 32 watt bulbs that emit 2,800 lumens (Sylvania) with a 16 h day/8 h night photoperiod ambient lab temperatures (22° C). The recovery period is 5 days at ambient lab temperatures. The plants have then been transferred to the greenhouse, then uncovered and cultured at its ambient temperatures (30° C day, 24° C night). The plants are allowed to grow for two weeks ambient temperatures. The visual selection of transgenic *G*. *max* roots has been performed with the enhanced green fluorescent protein (eGFP) reporter which is incorporated into the pRAP15 and pRAP17 vectors [50]. The eGFP reporting roots also possess the XTH43-OEor XTH43-RNAi cassette [48]. The eGFP reporter and expression cassette (OE or RNAi) each have their own promoter and terminator sequences. During this incubation period, K599 transfers the DNA cassettes that are located between the left and right borders of the pRAP15 and pRAP17 destination vectors into the root cell chromosomal DNA [53,54]. The result is a stable transformation event in the root somatic cell chromosome [58]. Roots subsequently develop from this transgenic cell located at the base of the shoot stock over a period of a few weeks, resulting in the generation of a genetically mosaic plant having a non-transgenic shoot with a transgenic root system. Consequently, in the experiments presented here, each individual transgenic root system is an independent transformant root. The transgenic plants have then been planted in a sandy (93.00% sand, 5.75% silt, and 1.25% clay) soil in SC10 Super cone-tainers (Stuewe and Sons^®^, Inc.) secured in RL98 trays (Stuewe and Sons^®^ Inc.). The plants then recover for two weeks prior to the start of the experiment at ambient greenhouse temperatures and photoperiod [50]. The cDNA has been confirmed to not contain genomic DNA by using PCR of cupin (Glyma.20G148300) which amplifies across an intron (S1 Table). The eGFP has been confirmed by performing PCR on cDNA from transgenic and non-transgenic roots (S1 Table). The functionality of the genetic constructs in their ability to overexpress the gene of interest (increase the relative transcript abundance of XTH43) or undergo RNAi (decrease the relative transcript abundance of XTH43) in *G*. *max* has been confirmed by RT-qPCR (Please refer to RT-qPCR subheading of the Materials section). The controls for the transgenic experiments are stated here as follows. As the controls for the experiments, the un-engineered pRAP15 or pRAP17 vectors have the *ccd*B gene. The *ccd*B gene is located in the position where, otherwise, XTH43 has been incorporated during the original cloning procedure. This feature makes the un-engineered pRAP15-*ccd*B (OE control) and pRAP17-*ccd*B (RNAi control) vectors suitable controls for any non-specific effects caused by gene overexpression or RNAi [48,50,59].

### The female index analysis of *H*. *glycines* parasitism

Prior experiments have shown XTH43 overexpression leads to a 70–89% decrease (p < 0.05) in *H*. *glycines* parasitism as measured by the community-accepted standard representation of the obtained data known as the female index (FI) [48,60]. The FI = (Nx/Ns) X 100, where Nx is the average number of females on the test cultivar and Ns is the average number of females on the standard susceptible cultivar [60]. In the experiments presented here, Nx is the pRAP17-XTH43 (RNAi) transformed root (XTH43-RNAi) and Ns is the pRAP17-*ccd*B control root (RNAi control) which has been calculated according to our prior published methods [50]. The FI has been calculated as cysts per mass of the whole root (wr) and also cysts per gram (pg) of root according to our published methods [50]. The wr analysis is how the data has been presented historically, not taking into consideration root performance [60]. The pg analysis accounts for possible altered root growth caused by the influence of the RNAi of XTH43. Three biological replicates have been made for the XTH43-RNAi and RNAi control with 10–20 individual transgenic plants serving as experimental replicates, each. A statistical analysis has been performed using the Mann–Whitney–Wilcoxon (MWW) Rank-Sum Test, p ≤ 0.05 cutoff [61,62]. The MWW Rank-Sum Test, as stated, is a nonparametric test of the null hypothesis not requiring the assumption of normal distributions [61].

### Histology

Histology has been done according to Klink et al. (2005) [63]. Staining has been done using safranin-O-fast green according to Klink et al. (2005) [63]. Microscopy has been performed on a Leica DM750 microscope using a Leica ICC50 E camera.

### Reverse transcriptase quantitative PCR (RT-qPCR) assessment of gene expression

The method of McNeece *et al*. (2019) has been followed for cDNA synthesis [50]. *G*. *max* root mRNA has been isolated using the UltraClean^®^ Plant RNA Isolation Kit (Mo Bio Laboratories^®^, Inc.) according to the manufacturer’s instructions. Genomic DNA has been removed from the mRNA with DNase I (Invitrogen^®^) according to the manufacturer’s instructions. The cDNA has been synthesized from RNA using SuperScript^®^ First Strand Synthesis System for RT-qPCR (Invitrogen^®^) with oligo d(T) as the primer according to the manufacturer’s instructions. Assessment of gene expression in *G*. *max* has been accomplished by RT-qPCR using Taqman^®^ 6-carboxyfluorescein (6-FAM) probes and Black Hole Quencher (BHQ1) (MWG Operon) (S1 Table) [50]. A ribosomal S21 protein coding gene (Glyma.15G147700) has been used as the RT-qPCR control [50]. The 2^-ΔΔ*C*^_T_ method of Livak and Schmittgen (2001) has been used to calculate the gene expression fold change caused by the genetic engineering event from three independent replicates [50,64]. The p-values have been calculated using a Student’s *t*-test for the replicated RT-qPCR reactions [65]. The results are significant if p ≤ 0.05.

### Preparation of cell wall material

The transgenic roots for XTH43-OE, XTH43-RNAi and their appropriate OE control and RNAi controls have been analyzed for differences in cell wall composition using uninfected roots for all analyses. The roots have been crushed in liquid nitrogen in an autoclaved and cooled mortar and pestle. After crushing, the samples have been kept at -80°C and freeze-dried (Labconco, Freezone^®^ 4.5 Plus). The sample weights have been recorded and the samples kept at 4°C until processed for further analyses.

### Removal of root extractives

A procedure of the NREL/TP-510-42619 standard has been followed in order to remove extractives from the root samples [66]. In this procedure, the samples have been wrapped in Kimwipes^®^ and stapled into 2 cm Kimwipes^®^ square packets. The samples have first been Soxhlet-extracted in water for 15 hours and subsequently air-dried overnight. The air-dried samples have been returned into Soxhlets and extracted in 95% ethanol for 24 hours. The samples have been kept at room temperature for two days to allow for the evaporation of ethanol. Additionally, the samples have then been dried in an oven at 50°C until reaching constant mass (Heratherm ^™^ OMS60, Thermo Fisher Scientific ^™^). The dry masses of the samples have been recorded before further processing, and samples stored in a desiccator until further processing.

### Removal of starch, protein, glycoprotein and pectin

Prior studies have shown that 1 gram of dry tissue of *G*. *max* roots contains 2.5 mg of protein and 25 mg of starch [67,68]. Based on these published measurements, to remove starch, weighed root samples have been treated with porcine pancreas α-amylase (Sigma-Aldrich^®^). The procedure has required the addition of 25 units of amylase (capable of releasing 25 mg of starch) per 1 gram of sample. Amylase has been diluted in sufficient volume of 100 mM Tris-HCl buffer (pH 7.0) to completely react with the starch present within the studied samples amount. The de-starching reaction has been performed at 37°C for 3 hours. Subsequently, the solution has been centrifuged at 10,000 rpm for 5 minutes and the supernatant discarded. To each gram of de-starched root samples, 0.025 mg of Pierce^™^ Protease (Thermo Fisher Scientific ^™^), capable of removing 2.5 mg of protein, along with sufficient amount of 50 mM acetic acid (pH 8.0) has been added to enzymatically react with the sample material with the reaction being carried out at 37°C for 4 hours. The solution has then been centrifuged at 4,000 rpm for 8 minutes, and the pellet has been washed with deionized water (10 ml). Upon removal of the supernatant, the pellet has been de-pectinated three times by boiling at 100° C in 50 mM EDTA (pH 6.8) for 15 minutes. The extracted pectin has been removed by centrifugation and then the de-pectinated samples have been further subjected to fractionation in 4% and 24% KOH solutions for extraction of loosely bound and tightly bound polysaccharides, respectively. This method removes glycoproteins [69]. Amyloid XyG has only been found as a food reserve in plant embryos. Consequently, Amyloid XyG is not believed to interfere with the results obtained from analyses of transgenic roots presented here [7].

### Polysaccharide fractionation

A procedure described by Nishikubo *et al*. (2011) [49] has been followed to perform polysaccharide fractionation. Loosely-bound hemicelluloses have been obtained by extracting the polysaccharides with three portions of 4% (w/v) KOH at room temperature on a rocker platform, first for 2 hours, then overnight, and finally for 2 minutes. The supernatants or extracts (loosely-bound polysaccharides) of each step have been combined and labeled as 4% KOH extracts. The remaining root tissues have been extracted in three portions of a solution containing 24% (w/v) KOH and 0.02% (w/v) sodium borohydride (NaBH_4_) in a similar manner (first for 2 hours, then overnight, and finally for 2 minutes). Three of these supernatant extracts (tightly-bound polysaccharides) have been combined and labeled as 24% KOH extracts. All KOH extractives have been neutralized with concentrated acetic acid on ice, and dialyzed in a dialysis tubing (Spectra/Pro6^®^ 1000 MWCO; Spectrum^®^) against water until the conductivity is lower than 1 mS. The dialyzed materials have been subjected to freeze-drying until reaching a constant mass. The final dry mass of the 4% and 24% KOH-extracts was recorded and used to determine % of loosely- and tightly-bound sugars in the samples, respectively.

### XyG quantification

The XyG content of the samples has been estimated by measuring VIS absorption at 660 nm on a microplate spectrometer (Epoch ^™^, Biotek^®^). The calibration curve was prepared from a series of tamarind (*Tamarindus indica*) standards (Megazyme^™^) in a concentration of 1,500, 1,000, 500, 250, 150 and 62.5 parts per million (ppm). Freeze-dried samples of 4% and 24% KOH-extracted polysaccharides have been diluted in water so to obtain a 660 nm peak in the calibration curve range (~8 μg/μl). The diluted samples have been heated at 80°C for 10 minutes. Lugol’s solution (Fisher Scientific^™^) has been diluted 10 times to obtain approximately 20 mM I_2_ and 60 mM KI solution. Then, 30 μl of each sample has been mixed with 15.1 μl of diluted Lugol’s solution and 151 μl of sodium sulfate solution (1.4 M) as per Kooiman (1957) [70]. Upon incubation for an hour at room temperature, the absorbance has been measured and XyG amounts relative to weight of freeze-dried sample expressed in percent (%). The difference in XyG amounts of 4% and 24% KOH extracts occurring between the transgenic and control samples have been assessed by analysis of variance (ANOVA) with post-hoc Tukey HSD test using SAS (SAS^®^ Institute) software.

### Molecular weight analysis by gel permeation chromatography (GPC)

The water diluted polysaccharide samples (~8 μg/μl) have been analyzed for molecular weight. Six dextran GPC analytical standards (Sigma-Aldrich^®^) in the range of 25 kDa to 1,400 kDa have been used for molecular weight calibration. The samples and the standards have been filtered through 0.2 μm filters and then analyzed by GPC using Refrectomax 520 RI (Thermo Fisher Scientific ^™^) detector and HPLC Finnigan Surveyor System (Thermo Fisher Scientific ^™^) controlled by Chromeleon ^™^ v.7 software (Thermo Scientific ^™^). The separation of MWs has been performed by Superose ^™^ 6 10/300 column (GE Healthcare ^™^), using water mobile phase at the flow rate of 0.2 ml/min (back pressures 277 psi) over a period of 120 minutes. The Superose ^™^ 6 10/300 column is designed for the fractionation range of M_p_ * 1000 to 3 × 10^5^ (dextrans) which covers the range of analyzed XyG [49]. In order to determine whether there was a difference between genetically modified and control root samples, GPC spectra have been compared by Principal Component Analysis (PCA) using the Origin 2018 software (version 95E).

### Weight average molecular weight (WAMW) calculation

Weight average molecular weight (WAMW) for each spectral profiles were calculated.

$$\text{WAMW}=\sum_{i=0}^{\infty }\frac{Hi\;Mi}{\sum Hi}$$

In the WAMW formula, *Mi*, represents molecular weight (kDa) fraction as a function of log for a very broad distributions of a linear molecular weight scale (standard dextrans). *Hi*, represents mass in i^th^ class of polymer in each degree of polymerization (DPs). Then, their sum over all classes give the total mass of the polymer [71,72]. WAMW values were compared among the samples via ANOVA Tukey test using SAS (SAS^®^ Institute) software.

## Results

### Transgenic root production

**Fig 1:**
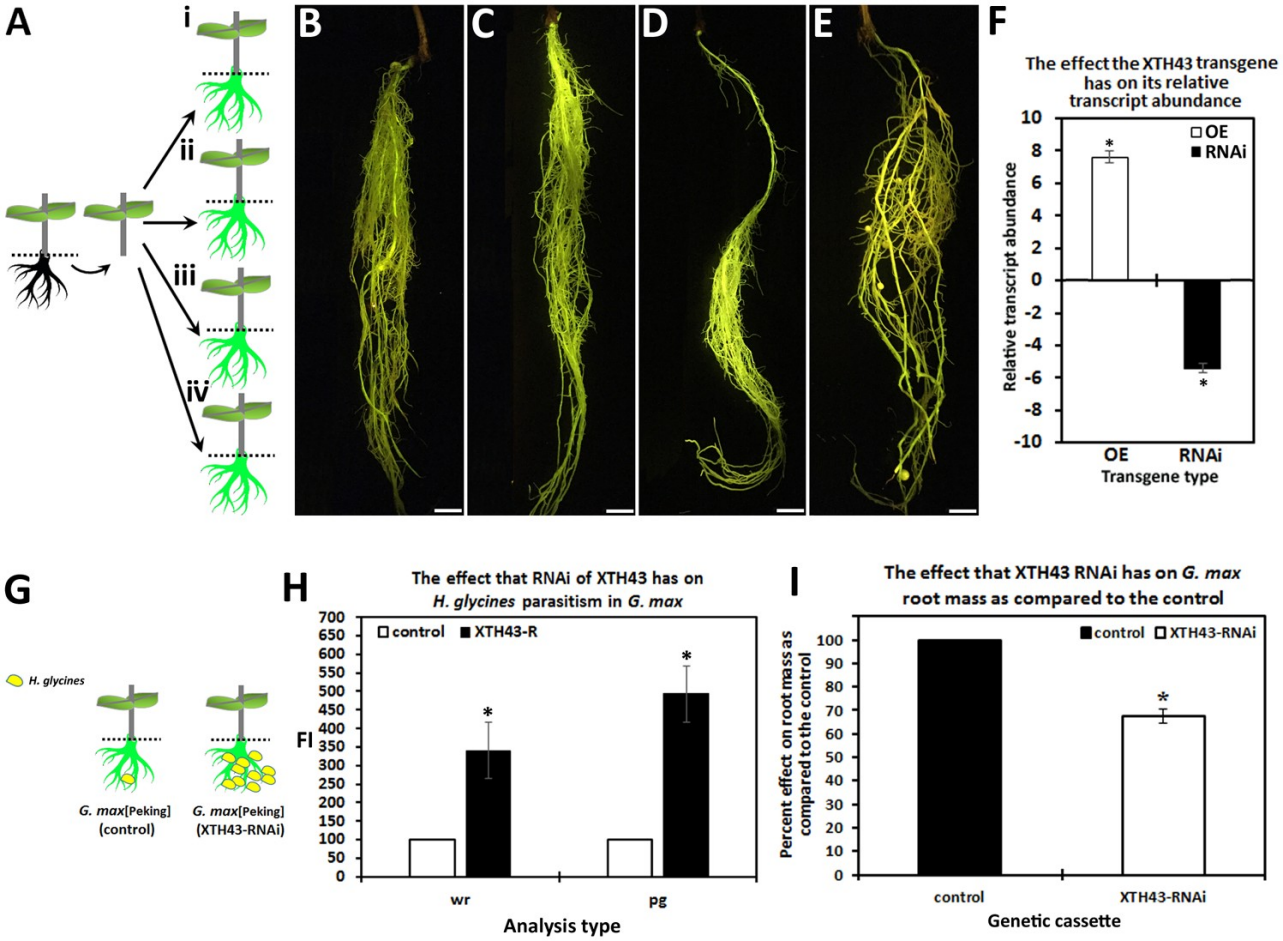
XTH43-RNAi impairs *H*. *glycines* parasitism, revealed by the female index (FI). **A**. *G*. *max* plant (left panel) prior to genetic engineering. Dashed line indicates where root removal occurs. Root removal (center panel) allows access for K599 to deliver the XTH43-OE or XTH43-RNAi transgene cassette. Right panel, the genetic constructs; **i**, *G*. *max*_[Peking/PI 548402]_-XTH43-RNAi; **ii**, *G*. *max*_[Peking/PI 548402]_-RNAi-control; **iii**, *G*. *max*_[Williams 82/PI 518671]_-XTH43-OE; **iv**, *G*. *max*_[Williams 82/PI 518671]_-OE-control after growth of new genetically engineered eGFP reporter-expressing roots. **B**. XTH43-OE in *G*. *max*_[Williams 82/PI 518671]_; **C**. pRAP15-control (OE control) in *G*. *max*_[Williams 82/PI 518671]_; **D**. XTH43-RNAi-expressing roots in *G*. *max*_[Peking/PI 548402]_; **E**. pRAP17-control (RNAi control) in *G*. *max*_[Peking/PI 548402]_. **F**. XTH43-OE RT-qPCR in *G*. *max*_[Williams 82/PI 518671]_ and XTH43-RNAi-expressing roots in *G*. *max*_[Peking/PI 548402]_ as compared to their respective controls. **F**. RT-qPCR confirms the increased XTH43 expression in the XTH43-OE root and decreased expression of XTH43 in the XTH43-RNAi root. **G**. The outcome of the infection of XTH43-RNAi root compared to the RNAi control showing more *H*. *glycines* in the XTH43 RNAi root compared to the control. The analyses determine the FI for the number of *H*. *glycines* per whole root (wr) mass and the number per gram (pg) of root tissue of XTH43-RNAi compared to the RNAi control (shown by the dashed line where the root was cut and weighed). **H**. the FI-generating experiments are run in triplicate with three independent biological replicates with p-values for OE (p = 0.0054) and RNAi (p = 0.0013) presented. Standard deviation is shown. * The results are statistically significant using the Mann–Whitney–Wilcoxon (MWW) Rank-Sum Test, p ≤0.05 cutoff (Mann and Whitney, 1947). **I**. Effect that the XTH43-RNAi transgene has on root mass as compared to the RNAi control (p = 0.0086). * statistically significant (p ≤0.05).

The constructs, *G*. *max* and experimental outcomes relating to the experiments are presented for clarity (Table 1; Fig 1). Statistical significance for all studies is set at p ≤0.05. The root phenotype for the transgenic plants and their respective controls are presented (Fig 1). XTH43 relative transcript abundance is determined in the XTH43-OE and XTH43-RNAi transgenic roots as compared to their respective controls (Fig 1). The approach used for the infection of the transgenic *G*. *max* genotype and anticipated outcome is presented (Fig 1). The FI assay in the XTH43-RNAi genetic roots show a statistically significant 3.41 fold increase for cysts per whole root (wr) compared to the RNAi control. Similarly, XTH43-RNAi leads to a statistically significant 4.93 fold increase in *H*. *glycines* FI compared to the RNAi control for cysts per gram (pg) of root. A noticeable difference in the FI occurs in the *H*. *glycines* analysis of cysts pg as compared to the cysts per wr, showing XTH43-RNAi statistically significantly decreases root mass (Fig 1). However, CW composition is the focus of this analysis.

**Table 1:** Constructs, *G*. *max* genotypes and experimental outcomes relating to the experiments.

Construct	*G*. *max* genotype	Relationship to *H*. *glycines*_[NL1-Rhg/HG-type 7/race 3]_
**XTH43-OE**	_**Williams 82/PI 518671**_	**resistant**
**pRAP15 (OE control)**	_**Williams 82/PI 518671**_	**susceptible**
**XTH43-RNAi**	_**Peking/PI 548402**_	**susceptible**
**pRAP17 (RNAi control)**	_**Peking/PI 548402**_	**resistant**

**Fig 2:**
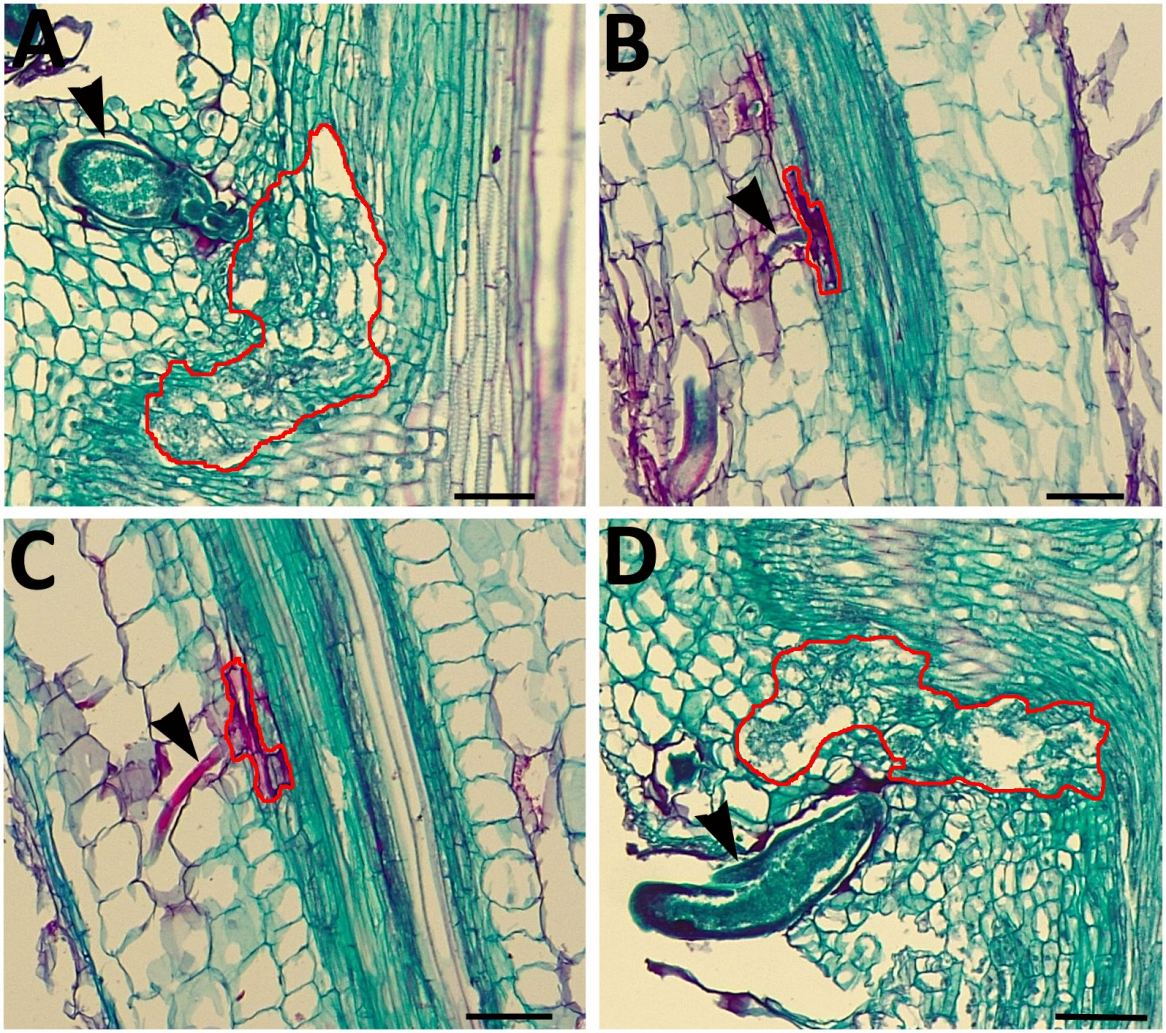
Histology of *H*. *glycines* parasitism 6 dpi in control and transgenic roots. **A**. Cytological section of the OE control in the *H*. *glycines*-susceptible genotype *G*. *max*_[Williams 82/PI 518671]_ showing *H*. *glycines* successfully parasitizing the root as evidenced by the production of a syncytium. **B**. Cytological section of the RNAi control in *H*. *glycines*-resistant *G*. *max*_[Peking/PI 548402]_ showing *H*. *glycines* unsuccessfully parasitizing the root as evidenced by its failure to produce a syncytium. **C**. Cytological section of an XTH43-OE root *H*. *glycines*-susceptible genotype *G*. *max*_[Williams 82/PI 518671]_ showing *H*. *glycines* where the syncytium failed to form. **D**. Cytological section of an XTH43-RNAi root in the *H*. *glycines*-resistant *G*. *max*_[Peking/PI 548402]_ showing *H*. *glycines* developing a syncytium. Black arrowhead, *H*. *glycines*; red line, syncytium boundary. Bar = 20 μμm.

Histological analyses of the OE control show the normal development of a syncytium (Fig 2A). In contrast, XTH43-OE shows the failure of *H*. *glycines* to produce a syncytium (Fig 2 and 2B). Histological analyses of the RNAi control show the normal resistant reaction (Fig 2C). In contrast, XTH43-RNAi shows the development of a syncytium that is comparable to that found in the RNAi control (Fig 2D).

### XTH43-OE: Total sugar and XyG as compared to its OE control

**Fig 3:**
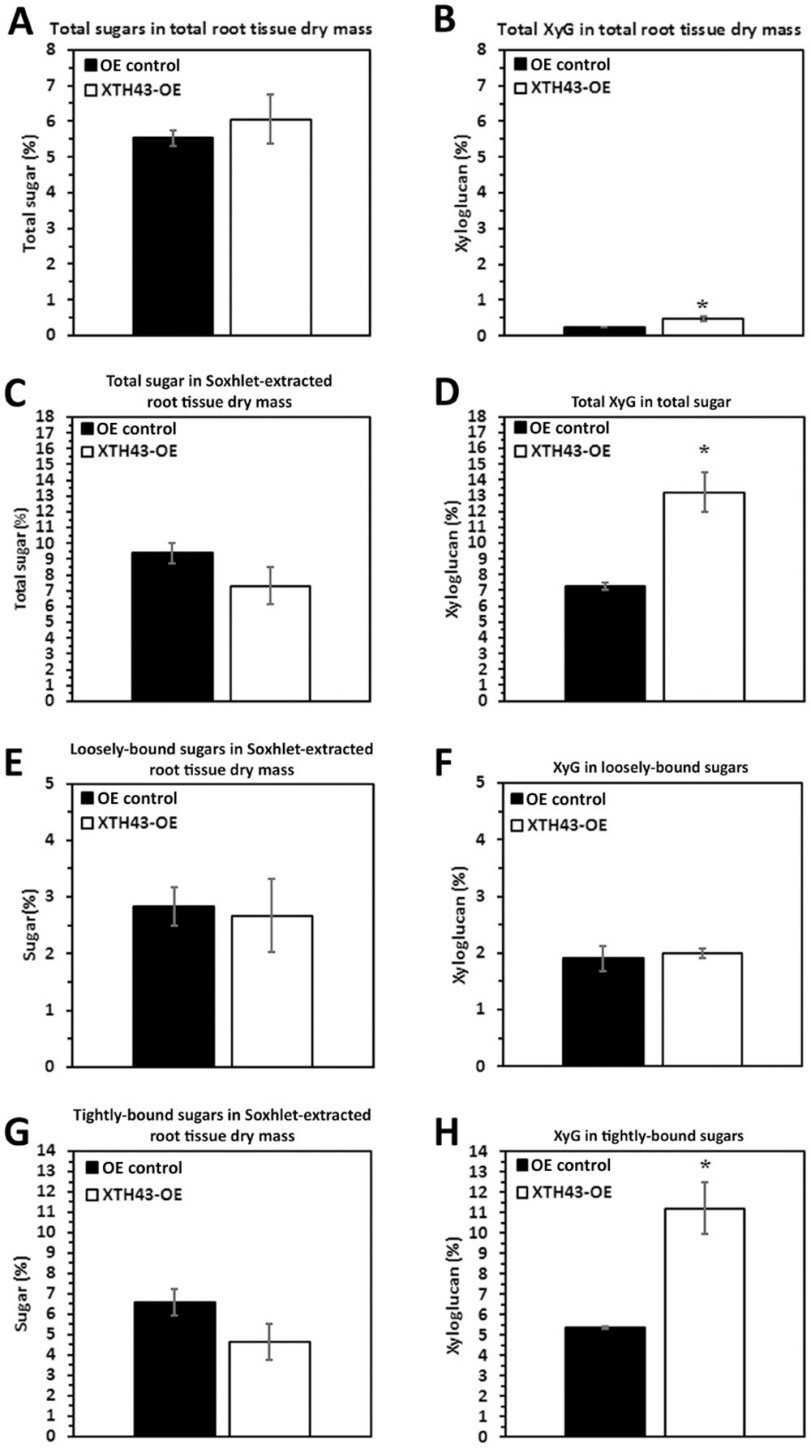
The amount (in percent) of total sugars and XyG in Soxhlet-extracted total sugars from *G*. *max* root total root tissue dry mass of XTH43-OE roots as compared to the OE control. **A**. Total sugars (p = 0.5107). **B**. Total XyG (p = 0.0123). **C**. Total sugar in Soxhlet-extracted root tissue dry mass (p = 0.1084). **D**. Total XyG in total sugar (p = 0.0183). **E**. Loosely-bound sugar in Soxhlet-extracted root tissue dry mass (p = 0.8337). **F**. XyG in loosely-bound sugar (p = 0.3602). **G**. Tightly-bound sugar in Soxhlet-extracted root tissue dry mass (p = 0.1446). **H**. Tightly-bound XyG in tightly-bound sugar (p = 0.0223). OE experiments are performed in the *H*. *glycines*-susceptible *G*. *max*_[Williams 82/PI 518671]_. RNAi experiments are performed in the *H*. *glycines*-resistant *G*. *max*_[Peking/PI 548402]_. (*) represents statistically significant difference p ≤ 0.05 calculated by t-test using Welch Two Sample t-test. Error bars represent standard error.

**Fig 4:**
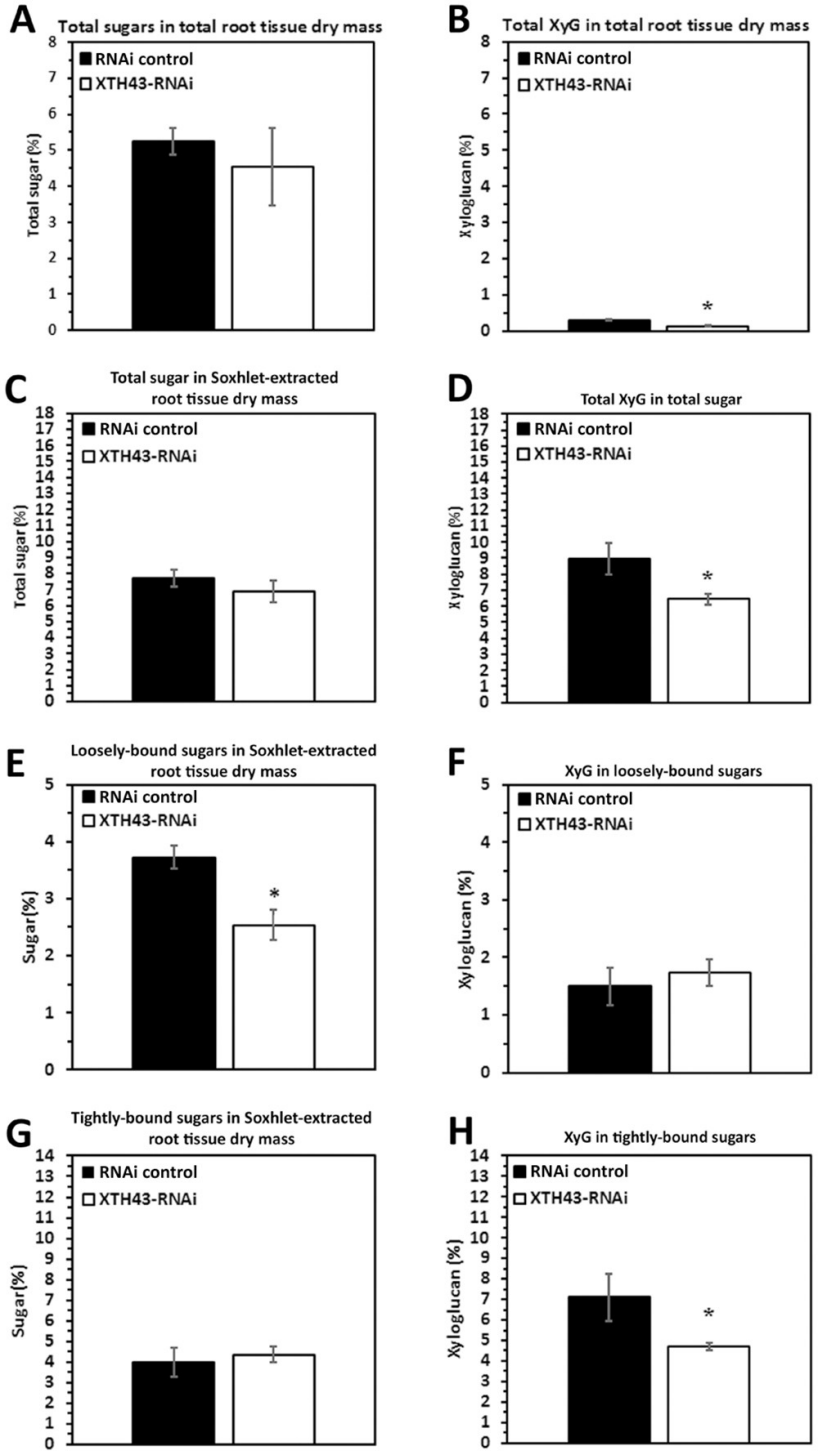
The amount (in percent) of total sugars and XyG in Soxhlet-extracted total sugars from *G*. *max* root total root tissue dry mass from XTH43-RNAi as compared to the RNAi control. **A**. Total sugar in total root tissue dry mass (p = 0.5631). **B**. Total XyG (p = 0.0213). **C**. The amount (in percent) of total sugars and XyG in Soxhlet-extracted total sugars (p = 0.1946). **D**. Total XyG in total sugar. **E**. Loosely-bound sugar in Soxhlet-extracted root tissue dry mass (p = 0.0237). **F**. XyG in loosely-bound sugar (p = 0.2977). **G**. Tightly-bound sugar in Soxhlet-extracted root tissue dry mass (p = 0.6673). **H**. XyG in tightly-bound sugar (p = 0.0355). RNAi experiments are performed in the *H*. *glycines*-resistant *G*. *max*_[Peking/PI 548402]_. (*) represents statistically significant difference p ≤ 0.05 calculated by t-test using Welch Two Sample t-test. Error bars represent standard error.

The CW analyses focuses in on root CW sugar and XyG composition in the XTH43-OE and XTH43-RNAi transgenic roots compared to their respective OE control and RNAi control (Figs 3 and 4). No significant difference in the relative amounts of total sugars (calculated as a sum of loosely and tightly-bound sugars) in total root tissue dry mass is found in comparisons of XTH43-OE to its OE control (Fig 3A). In contrast, XyG relative amount in total root tissue dry mass indicate a significantly 2.04-fold higher percent of XyG in XTH43-OE as compared to the OE control (Fig 3B). The result indicates that while no overall difference in total sugar content occurs in XTH43-OE samples as compared to the OE control, XyG content is increased.

The analysis then examines the relative amount (in percent) of total sugars and XyG in Soxhlet-extracted root material. The amount of total sugars in XTH43-OE transgenic roots is comparable to the OE control roots (Fig 3C). Among the XTH43-OE roots, the analyses identify a statistically significantly 1.81-fold higher level of XyG in the sugars obtained from Soxhlet-extracted samples as compared to the OE control roots (Fig 3D). The results identify a statistically significant difference in XyG occurring in XTH43-OE as compared to its OE control, and no difference in total sugars.

Designed experiments allow for the differentiation of the relative amounts of loosely-bound sugars (obtained through 4% KOH extraction) and tightly-bound sugars (obtained through 24% KOH extraction) and their XyG component in XTH43-OE transgenic roots and OE control to provide important insight into the specific role of XTH43 in relation to CW composition. The loosely-bound sugar amounts from XTH43-OE, calculated relative to dry-mass of Soxhlet-extracted roots are not significantly different as compared to loosely-bound sugars in the OE control (Fig 3E). Overall, similar values for all relative amounts are obtained in XTH43-OE XyG being comparable to the OE control and are not statistically significant for the percentage of XyG as a subset of loosely-bound sugars (Fig 3F). Therefore, the differences in XyG from the prior analyses are not originating from the loosely-bound component.

The percentage of tightly-bound sugars are determined from XTH43-OE and their OE control, respectively. Analyses of tightly-bound sugars from XTH43-OE roots, expressed as the relative amount of Soxhlet-extracted root tissue dry mass, are not statistically significantly different as compared to its OE control (Fig 3G). The relative amounts of tightly-bound sugars in the XTH43-OE are not significantly different from the OE control. In contrast, the percentage of XyG among the tightly-bound sugars are significantly different due to XTH43-OE (Fig 3H). The analyses of XTH43-OE tightly-bound sugar samples identify a statistically significant 2.09-fold higher amount of XyG as compared to the OE control roots. Consequently, the difference in XyG identified in the analysis of total dry mass appears to be coming from an increase in XyG and the specific type is tightly-bound.

### XTH43-RNAi: Total sugar and XyG as compared to its pRAP17-control

In RNAi-based studies, the analyses of total sugar in total root tissue dry mass in the XTH43-RNAi transgenic roots is not statistically significantly different from the RNAi control transgenic roots (Fig 4A). As expected, the calculated XyG in total root tissue dry mass for the XTH43-RNAi root samples shows a statistically significant -1.56 fold decrease in the relative amount of XyG as compared to the RNAi control transgenic roots (Fig 4B). This result is the expected opposite outcome found for the XTH43-OE experiment.

The analysis of the relative amount (in percent) of total sugars and XyG in Soxhlet-extracted root tissues (obtained as sum of 4% and 24% KOH extracts) from the XTH43-RNAi transgenic roots reveal that the XTH43-RNAi roots are not statistically significantly different from the RNAi control transgenic roots (Fig 4C). In contrast, there is a statistically significantly -1.39-fold lower level of XyG relative amounts in total sugar in the XTH43-RNAi roots as compared to the RNAi control root samples (Fig 4D). The result is the expected opposite outcome observed in the XTH43-OE experiment.

The relative amounts of loosely-bound sugars in root tissue dry mass are determined from the XTH43-RNAi transgenic roots and its respective RNAi control (Fig 4E). The study shows a significantly -1.47-fold lower relative amount of loosely-bound sugars in Soxhlet-extracted XTH43-RNAi tissue than its RNAi control. However, when examining the XyG content, the XTH43-RNAi transgenic roots have a XyG content that is not statistically different from the RNAi control (Fig 4F). The results show, compositionally, XTH43-RNAi does not have an effect on the relative XyG amount in loosely-bound sugars. Therefore, it appears the decrease in relative amounts of XyG found earlier in the total root dry mass and total sugar analyses (Fig 4B and 4D) are having an effect on the loosely-bound fraction of CW polysaccharides (Fig 4E). Analyses proceeded to determine whether the decrease in XyG observed earlier is due to a decrease in the tightly-bound fraction.

The relative amount of tightly-bound sugars in the Soxhlet-extracted root tissue in the XTH43-RNAi roots is not statistically significantly different from the RNAi control (Fig 4G). In contrast, the relative amount of XyG in tightly-bound sugars of XTH43-RNAi sample are significantly -1.51-fold lower as compared to the RNAi control (Fig 4H). The outcomes from the XTH43-OE and XTH43-RNAi analyses are largely inversely related to each other as expected.

### GPC analysis of loosely-bound sugars in XTH43 overexpression and RNAi roots

The previous analyses could not determine whether there are differences not only in the amount, but also in the molecular masses of the sugars in the XTH43-OE and XTH43-RNAi roots as compared to their respective controls. GPC in conjunction with PCA make those determinations. Dextran standard controls ranging from 25 kDa to 1,400 kDa determine mass distribution and stability of the instrument performance after each GPC analyses run, in consistent spectra and clear peak separations of the standards demonstrating that the instrument performs consistently and stably throughout the sample analysis procedure (S1 Fig).

**Fig 5:**
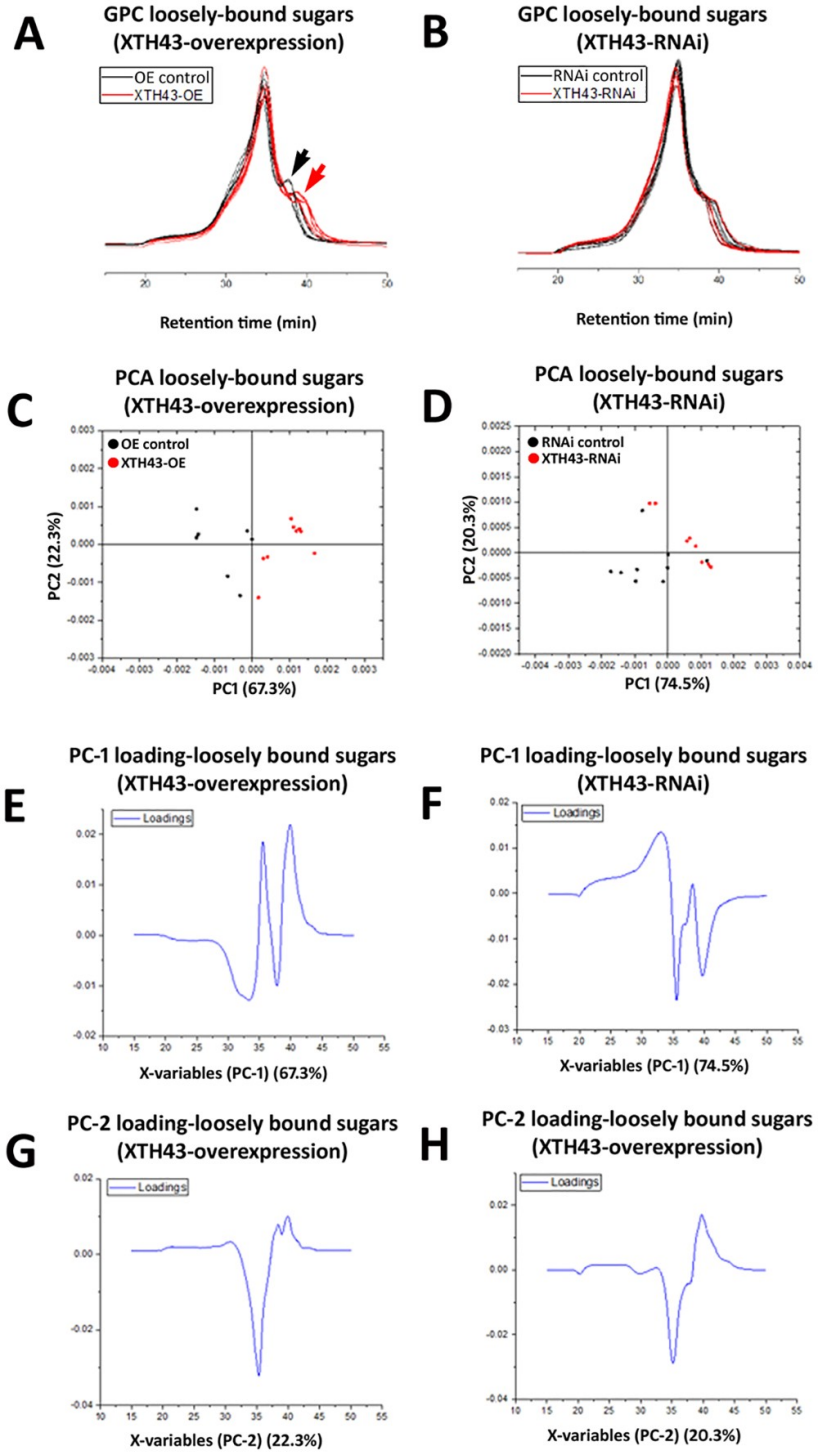
GPC and PCA analysis of loosely-bound sugars in OE control compared to XTH43 samples and RNAi control compared to XTH43-RNAi samples. **A**. Area normalized chromatogram of loosely-bound sugars in OE control (black) and XTH43-OE (red) samples. **B**. Area normalized chromatogram of loosely-bound sugars in RNAi control (black) and XTH43-RNAi (red) samples. **C**. PC-1/PC-2 score plot of OE control (black) and XTH43-OE sample spectra of loosely-bound sugars. **D**. PC-1/PC-2 score plot of RNAi control (black) and XTH43-RNAi sample spectra of loosely-bound sugars. **E**. PC-1 (OE control) loading. **F**. PC-1 (RNAi control) loading. **G**. PC-2 (XTH43-OE) loading. **H**. PC-2 (XTH43-RNAi) loading. OE experiments are performed in the *H*. *glycines*-susceptible *G*. *max*_[Williams 82/PI 518671]_. RNAi experiments are performed in the *H*. *glycines*-resistant *G*. *max*_[Peking/PI 548402]_.

GPC analyses of loosely-bound sugars from XTH43-OE and OE control roots reveal similar spectral profiles, comparable in elution times of 1,400 kDa MW dextran standards (20–40 min). However, a major difference occurring between these two sample types (XTH43-OE and OE control roots) occurs in the MW region of 35–40 min elution time, with XTH43-OE (red chromatograms) showing lower MW peaks, i.e. later elution times, than the OE control samples (black chromatograms) in this region (Fig 5A). The PCA analysis of spectra in 15–50 min elution time region show a clear separation of the XTH43-OE and OE controls by PC-1. The positive loadings of PC-1 indicate that XTH43-OE samples are characterized by overall higher relative amounts of loosely-bound sugars eluting at 35.5 min. It confirms the occurrence of a shift in spectra with peaks at 40 min elution belonging to XTH43-OE and somewhat larger MW polymers eluting at 37.5 min being characteristic of OE control (Fig 5E, **red and black arrow**). The PC-1 loadings also point out to the shoulder of the OE control, occurring at 33 min of elution time.

GPC analyses of XTH43-RNAi and pRAP17-control root loosely-bound sugars also reveal overall similar spectral patterns from the XTH43-RNAi (red chromatograms) and RNAi control roots (black chromatograms), with elution times between 30 and 45 minutes (Fig 5D). The differences between spectra of XTH43-RNAi and RNAi control roots are once again mainly explained by PC-1 scores. However, in this analysis, the RNAi samples show lower relative amounts of polymers eluting at 36 min, and the higher amounts of polymers eluting within a spectral shoulder with 33 min elution time. The peak at 40 min elution, in this case, is characteristic mainly of the RNAi control.

### WAMW of loosely-bound sugars in XTH43-OE and XTH43-RNAi roots

The differences in spectral molecular weight are examined further in analyses of the WAMW (Materials section). The GPC analyses are in agreement with the statistical analysis obtained through the WAMW study. A statistically significantly -8.87% lower MW exists for the loosely-bound sugar samples isolated from the XTH43-OE root, as compared to the RNAi control (Table 2). The WAMW analysis for the loosely-bound sugars confirm higher MW sugars occurring in the XTH43-RNAi root samples. However, the results are slightly above the threshold of significance (Table 2).

**Table 2:** Characterization of weight average molecular weight (WAMW) of extracted bound sugars in XTH43 transgenics (OE, RNAi) and their respective control roots.

	Loosely bound sugars (OE)	Loosely bound sugars (RNAi)	Tightly bound sugars (OE)	Tightly bound sugars (RNAi)
**XTH43**	**741.69 ±9.10**	**775.35 ±4.64**	**860.94 ±10.57**	**916.75 ±8.13**
**control**	**813.86 ±5.17**	**760.63 ±6.05**	**931.34 ±15.22**	**846.80 ±5.90**
**p-value**	**0.0012**	**0.063**	**0.0096**	**0.0011**

### GPC analysis of tightly-bound sugars in the XTH43 overexpressing and RNAi roots

**Fig 6:**
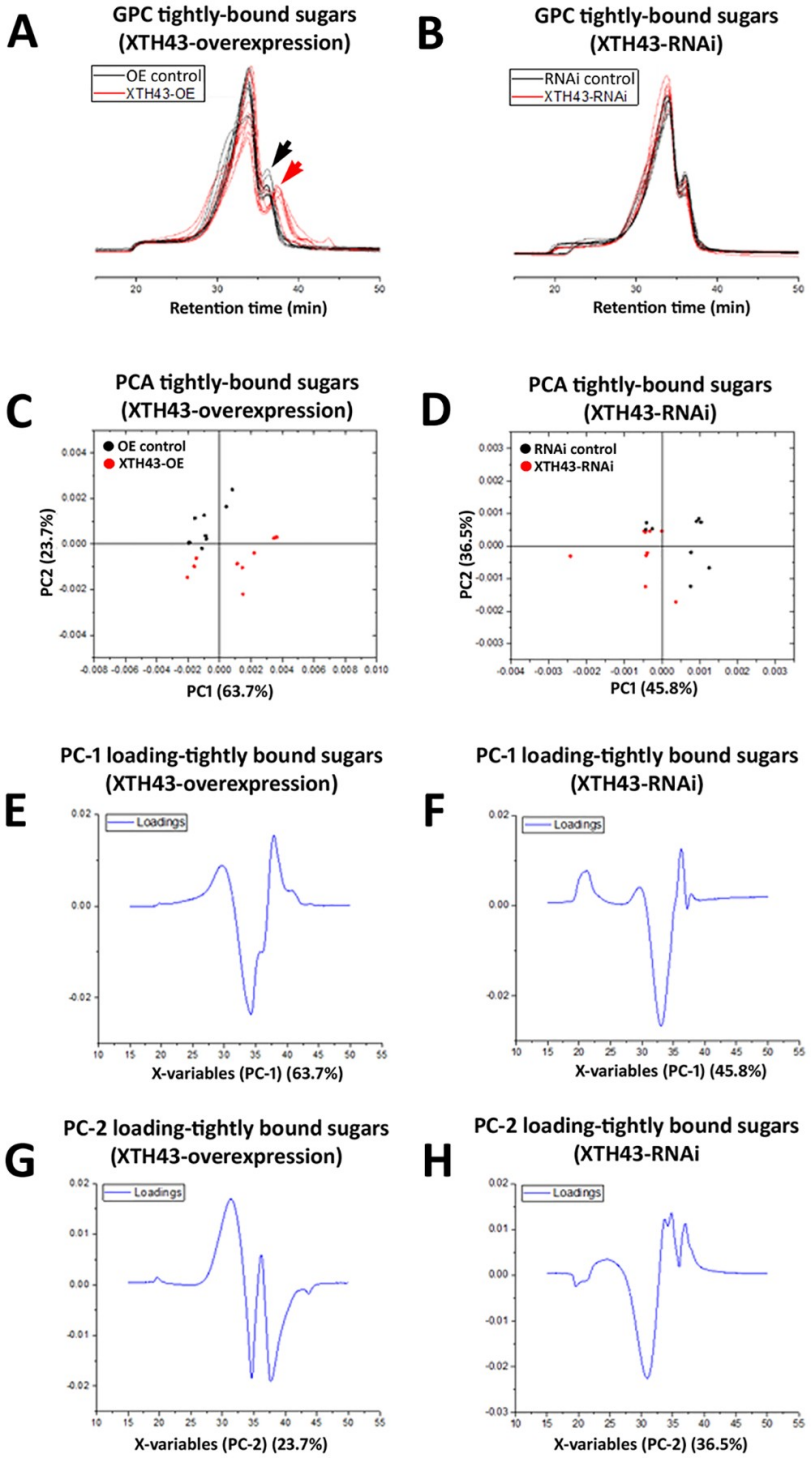
GPC and PCA analysis of tightly-bound sugars in RNAi control compared to XTH43 samples and RNAi control compared to XTH43-RNAi samples. **A**. Area normalized chromatogram of tightly-bound sugars in OE control (black) and XTH43-OE (red) samples. **B**. Area normalized chromatogram of tightly-bound sugars in RNAi control (black) and XTH43-RNAi (red) samples. **C**. PC-1/PC-2 score plot of OE control (black) and XTH43-OE sample spectra of tightly-bound sugars. **D**. PC-1/PC-2 score plot of RNAi control (black) and XTH43-RNAi sample spectra of tightly-bound sugars. **E**. PC-1 (OE control) loading. **F**. PC-1 (RNAi control) loading. **G**. PC-2 (XTH43-OE) loading. **H**. PC-2 (XTH43-RNAi) loading. OE experiments are performed in the *H*. *glycines*-susceptible *G*. *max*_[Williams 82/PI 518671]_. RNAi experiments are performed in the *H*. *glycines*-resistant *G*. *max*_[Peking/PI 548402]_.

A GPC analysis of tightly-bound sugars of XTH43-OE and OE control root samples show slight differences occurring between the replicate runs, despite the MeOH peak (internal standard) remaining at a constant elution time. Interestingly, and regardless of these slight differences, the tightly-bound sugars of both XTH43-OE and OE control samples elute faster than the loosely-bound sugar extractions (Fig 6A and 6B). The results indicate an expected overall higher tightly-bound sugar MW than loosely-bound sugars.

Tightly-bound sugars (red chromatograms) extracted from the XTH43-OE roots elute later than the OE control tightly-bound (black chromatograms) in 35–40 min elution time region, signifying the shorter tightly-bound sugars and therefore altered CW structural architecture due to XTH43-OE. These spectral differences are confirmed by PCA analyses, with PC-2 scores distinguishing between the sample groups, and assigning higher relative amounts of peaks with elution time of 34.5 min and the latest eluted peak at 37.5 min to XTH43-OE samples. The PC-2 loadings also points out to the shoulder at 31 min and 36.5 min elution peak being characteristic of the OE control samples (Fig 6).

GPC analysis of XTH43-RNAi tightly-bound sugars reveal a similar overall spectral pattern to the RNAi control (Fig 6). PCA show PC-1 loadings point out to the higher relative amounts of sugars eluting at 33 min mainly in XTH43-RNAi root samples. PC-2 loadings mainly explain the differences in relative amounts of shoulder peaks at 31 min elution, without giving a clear insight into the differences occurring between samples as related to the MW, as expected.

### WAMW analysis of tightly-bound sugars in XTH43 overexpression and RNAi roots

WAMW analyses confirm the GPC results. The WAMW show the tightly-bound sugars having a statistically significantly -7.56% lower MW occurring between the XTH43-OE and OE control samples (Table 2). The WAMW calculations of XTH43-RNAi root samples show a statistically significantly 7.63% higher MW in comparison to the RNAi control (Table 2). This result indicates XTH43-RNAi lengthens the tightly-bound sugars, expected from the higher relative amounts of sugars in the XTH43-RNAi root samples indicated by the PC-1 of the spectral analysis. Overall, for tightly-bound sugars, XTH43-RNAi yields the opposite effect on polysaccharide chain length than XTH43-OE.

### Direct comparison of the relative amounts of sugars and XyG occurring between the control samples of different genotypes

**Fig 7:**
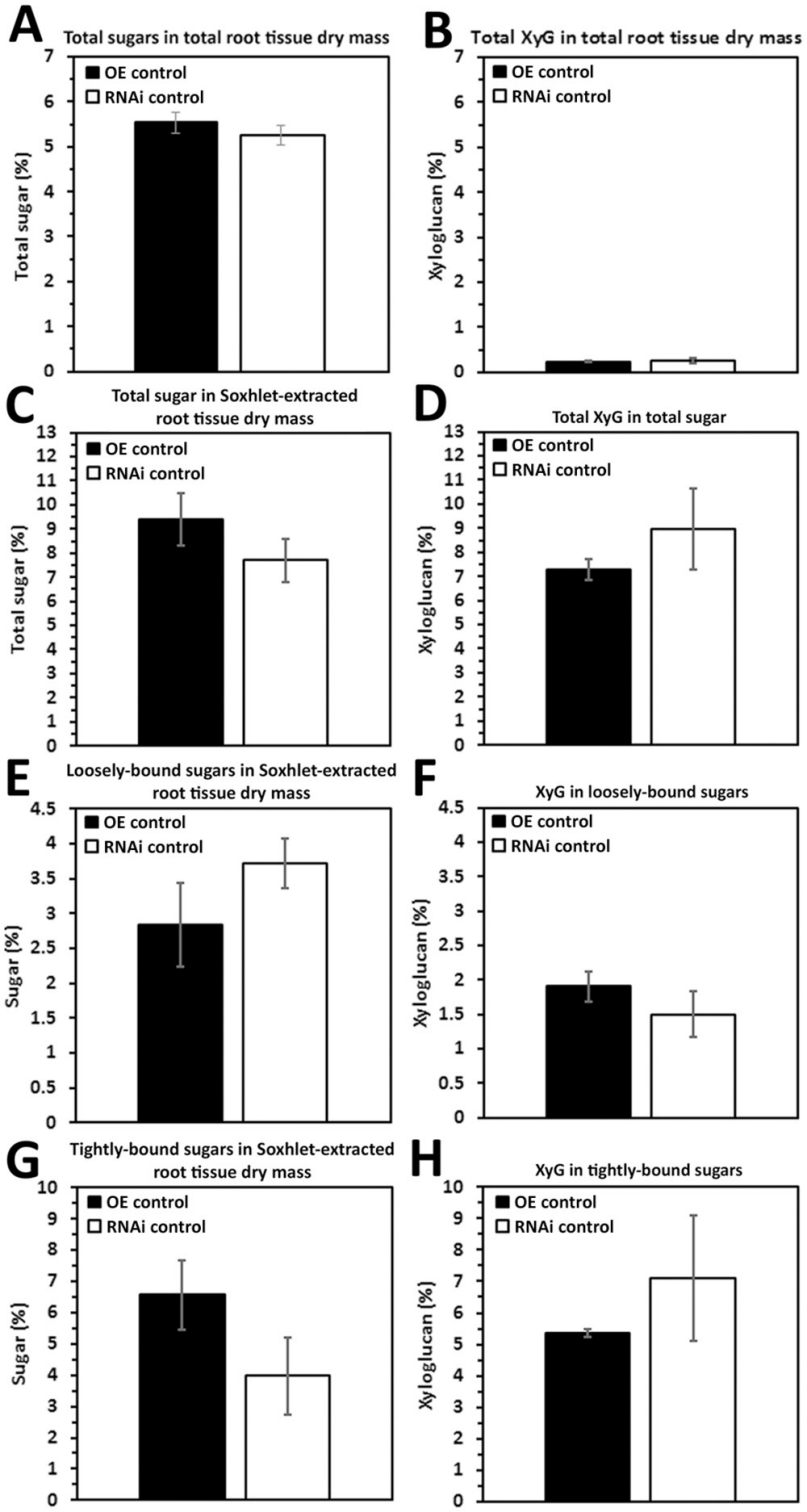
Direct comparisons made between the OE control and RNAi controls. **A**. Total sugars in total root tissue dry mass (p = 0.5374). B. Total XyG in total root tissue dry mass (p = 0.8323). C. Total sugar in Soxhlet-extracted root tissue dry mass (p = 0.1070). **D**. Total XyG in total sugar (p = 0.1678). E. Loosely-bound sugar in Soxhlet-extracted root tissue dry mass (p = 0.0920). **F**. XyG in loosely-bound sugar (0.3626). **G**. Tightly-bound sugar in Soxhlet-extracted root tissue dry mass (p = 0.0534). **H**. XyG in tightly-bound sugar (p = 0.2070). (*) represents statistically significant difference p ≤ 0.05 calculated by t-test using Welch Two Sample t-test. The results have are not statistically significant between the two genotypes. Error bars represent standard error. OE experiments are performed in the *H*. *glycines*-susceptible *G*. *max*_[Williams 82/PI 518671]_. RNAi experiments are performed in the *H*. *glycines*-resistant *G*. *max*_[Peking/PI 548402]_.

The next question is whether the two *G*. *max* genotypes are appreciably different in CW composition prior to *H*. *glycines* infection. Statistically significant differences do not occur between any comparable values of relative amounts of sugar per root tissue dry mass of OE control and RNAi control roots (Fig 7A, 7C, 7E and 7G). Similarly, the relative amounts of XyG in any of the sugar extractions of control samples did not differ statistically (Fig 7B, 7D, 7F and 7H). These results support the validity of using *G*. *max*_[Williams 82/PI 518671]_ and *G*. *max*_[Peking/PI 548402]_ as comparable genotypes for examining chemical differences in transgenically altered roots. However, these experiments do not account for the length of sugar chains which could be determined by GPC because it is possible that while the amounts of sugars and XyG are the same, the relative chain lengths may differ. These experiments are presented later.

### Direct comparison of the relative amounts of sugars and XyG occurring between XTH43-OE and XTH43-RNAi transgenic root samples

**Fig 8:**
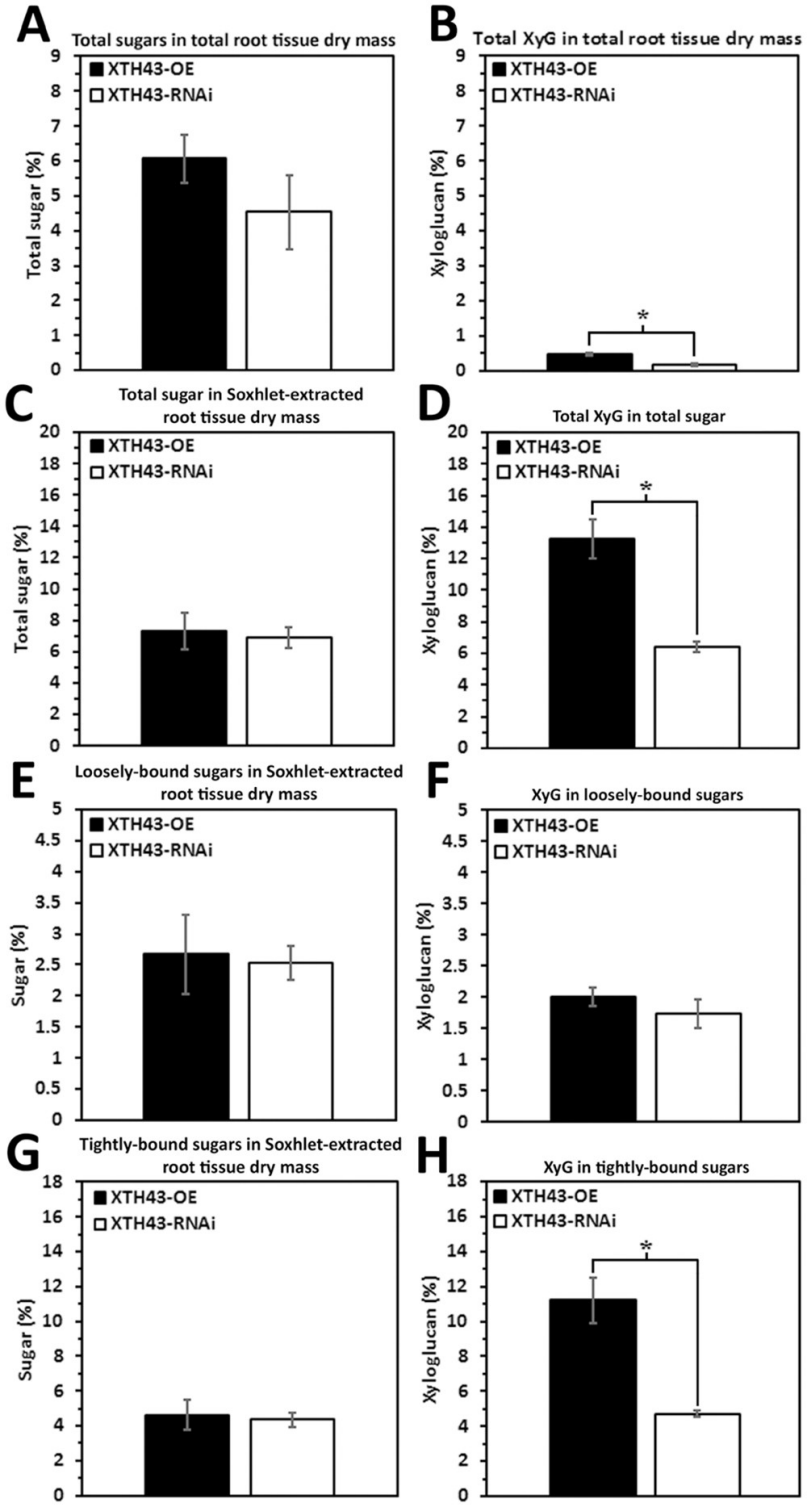
Direct comparisons made between the XTH43-OE and XTH43-RNAi. **A**. Total sugars in total root tissue dry mass (p = 0.2966). **B**. Total XyG in total root tissue dry mass (p = 0.0090). **C**. Total sugar in Soxhlet-extracted root tissue dry mass (p = 0.7742). **D**. Total XyG in total sugar (p = 0.0063). **E**. Loosely-bound sugar in Soxhlet-extracted root tissue dry mass (p = 0.8542). **F**. XyG in loosely-bound sugar (p = 0.3482). **G**. Tightly-bound sugar in Soxhlet-extracted root tissue dry mass (p = 0.7799). **H**. XyG in tightly-bound sugar (p = 0.0074). (*) represents statistically significant difference p ≤ 0.05 calculated by t-test using Welch Two Sample t-test. The results are not statistically significant between the two genotypes. Error bars represent standard error. OE experiments are performed in the *H*. *glycines*-susceptible *G*. *max*_[Williams 82/PI 518671]_. RNAi experiments are performed in the *H*. *glycines*-resistant *G*. *max*_[Peking/PI 548402]_.

It is hypothesized that XTH43-OE leads to sugar and/or XyG content exceeding transgenic roots undergoing XTH43-RNAi. No statistically significant differences are found regarding the relative amounts of sugars that are present in root tissue dry mass (Fig 8A, 8C, 8E and 8G). However, the relative amounts of XyG in total root tissue dry mass, Soxhlet-extracted root tissue, and tightly-bound sugar extracts are significantly higher in direct comparisons of XTH43-OE to XTH43-RNAi (Fig 8B, 8D and 8H, **respectively**). The relative amount of XyG in loosely-bound sugars is not found to be significantly different (Fig 8F). These results demonstrate that the statistically significant differences in XyG are caused by the XTH43-OE and XTH43-RNAi and not by compositional variances of the *G*. *max*_[Williams 82/PI 518671]_ and *G*. *max*_[Peking/PI 548402]_ genotypes.

### Direct GPC analyses of loosely-bound sugars

**Fig 9:**
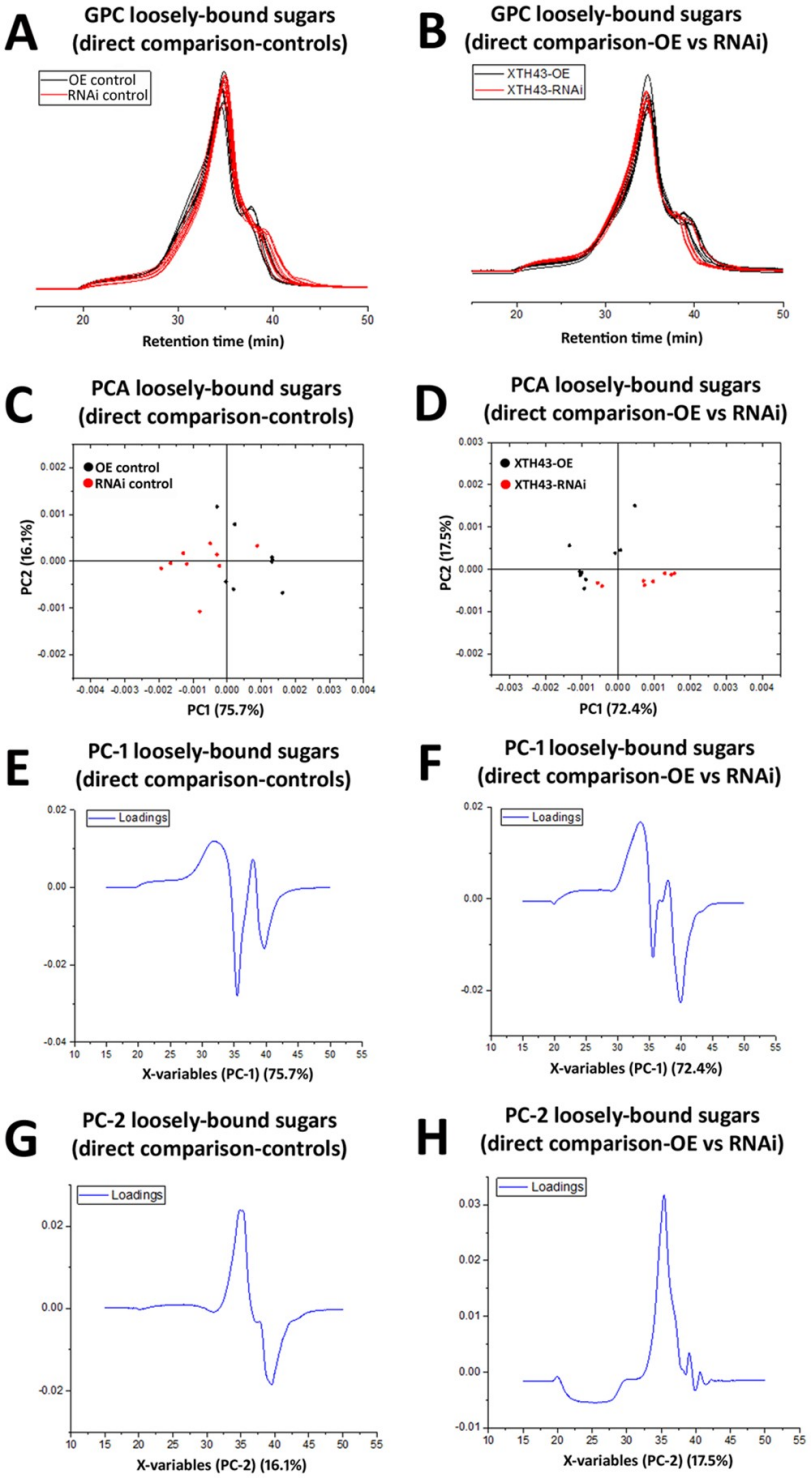
Direct GPC and PCA analysis of loosely-bound sugars in OE control compared to RNAi control and XTH43-OE compared to XTH43-RNAi samples. **A**. Area normalized chromatogram of loosely-bound sugars in OE control (black) and RNAi control (red) samples. **B**. Area normalized chromatogram of loosely-bound sugars in XTH43 (black) and RNAi control (red) samples. **C**. PC-1/PC-2 score plot of OE control (black) and RNAi control sample spectra of loosely-bound sugars. **D**. PC-1/PC-2 score plot of XTH43-OE (black) and XTH43-RNAi sample spectra of loosely-bound sugars. **E**. PC-1 (OE control) loading. **F**. PC-1 (XTH43-OE) loading. **G**. PC-2 (RNAi control) loading. **H**. PC-2 (XTH43-RNAi) loading. OE experiments are performed in the *H*. *glycines*-susceptible *G*. *max*_[Williams 82/PI 518671]_. RNAi experiments are performed in the *H*. *glycines*-resistant *G*. *max*_[Peking/PI 548402]_.

No differences in loosely-bound sugars are anticipated since no statistically significant differences in the sugar relative amounts occur between the OE control and RNAi control roots (Fig 7). As expected, loosely-bound sugars of the OE control and RNAi control are not statistically significantly different in sugar chain length (Fig 9). The differences identified by PCA are assigned to the amounts of the sugars in the shoulder eluting around 30 min and in the peak eluting at 40 minutes that are obvious in both types of samples. The peak shift at 37.5 min is not emphasized by the PC1 and PC2 loadings, as this peak is common only to a couple of the OE control samples. Direct comparisons examine loosely-bound sugars occurring between the XTH43-OE and XTH43-RNAi roots. However, that MW difference occurring between the XTH43-OE and XTH43-RNAi roots is not obvious (Fig 9). Furthermore, the samples are, similarly to the OE and RNAi controls, grouped in a narrow range of the PCA scores graph origin, and PCA mainly differentiated the outlier samples (Fig 9).

### Direct GPC analyses of tightly-bound sugars

**Fig 10:**
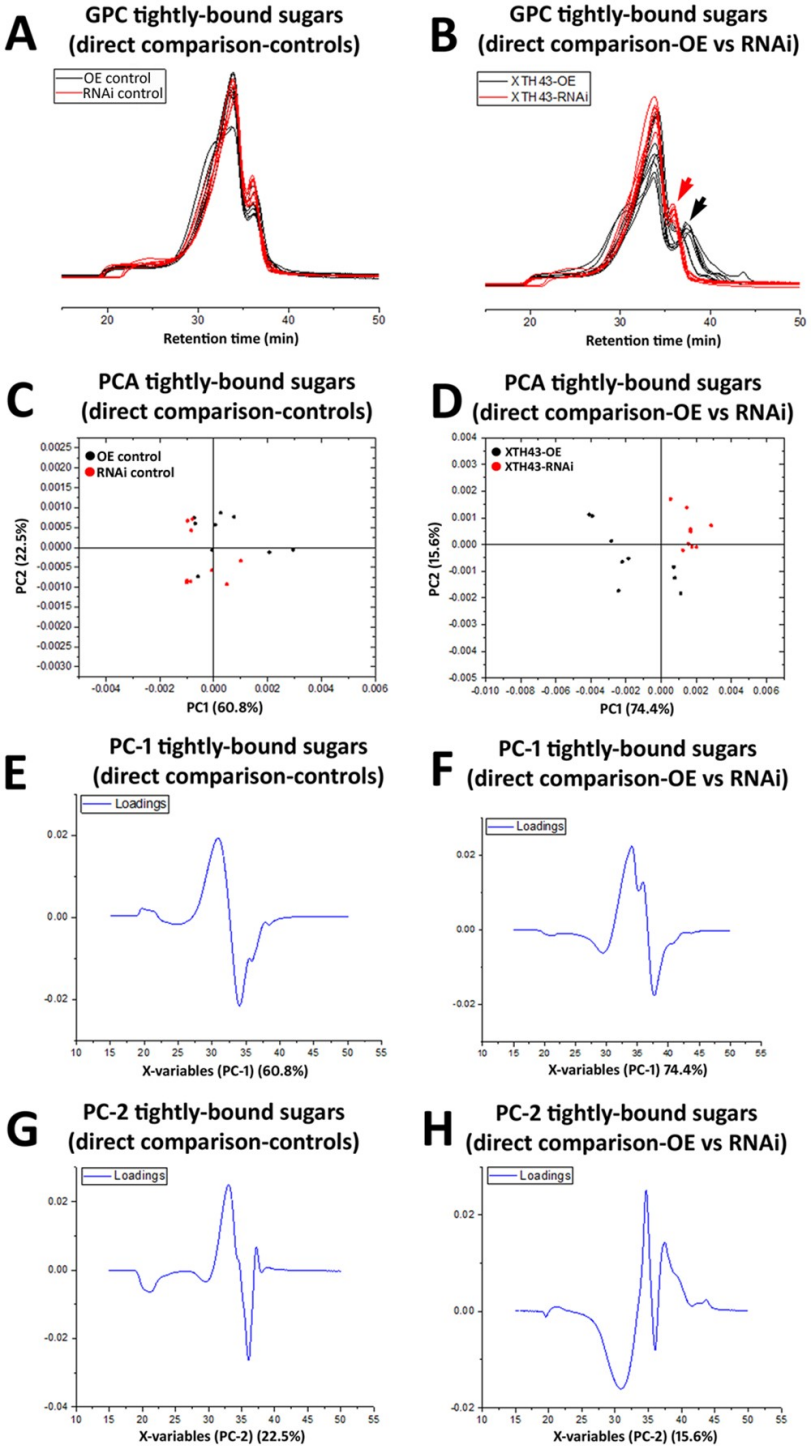
Direct GPC and PCA analysis of tightly-bound sugars in OE control compared to RNAi controls and XTH43-OE compared to XTH43-RNAi samples. **A**. Area normalized chromatogram of tightly-bound sugars in OE control (black) and RNAi control (red) samples. **B**. Area normalized chromatogram of tightly-bound sugars in XTH43 (black) and XTH43-RNAi-control (red) samples. **C**. PC-1/PC-2 score plot of OE control (black) and RNAi control sample spectra of tightly-bound sugars. **D**. PC-1/PC-2 score plot of XTH43-OE (black) and XTH43-RNAi sample spectra of tightly-bound sugars. **E**. PC-1 (OE control) loading. **F**. PC-1 (XTH43-OE) loading. **G**. PC-2 (RNAi control) loading. **H**. PC-2 (XTH43-RNAi) loading. OE experiments are performed in the *H*. *glycines*-susceptible *G*. *max*_[Williams 82/PI 518671]_. RNAi experiments are performed in the *H*. *glycines*-resistant *G*. *max*_[Peking/PI 548402]_.

The direct GPC analyses of tightly-bound sugars is similar for the OE control and RNAi control. In contrast, GPC identified a shift in spectra of tightly-bound sugars of XTH43-OE as compared to XTH43-RNAi, grouping the RNAi samples in one PCA quadrant, and showing a peak at 36 min in PC-1 and 35 min in PC-2 loadings graphs to be more prevalent in RNAi samples (Fig 10). This spectra likely confirms the differences in XyG content found in direct comparisons of XTH43-OE and XTH43-RNAi (Fig 8).

### Direct WAMW analyses of OE control and RNAi control samples

WAMW analyses compare the OE control directly to the RNAi control. The analyses of the loosely-bound sugars reveal a statistically significant difference of 6.54% in the OE control compared to the RNAi control (Table 3). A statistically significant difference of 9.08% exists in the OE control compared directly to the RNAi control for tightly-bound sugars (Table 3).

**Table 3:** Comparison of WAMW of extracted sugars.

**Comparison of WAMW of extracted bound sugars in OE control and RNAi control roots**		
	**Loosely bound sugars**	**Tightly bound sugars**
**OE control**	**813.86 ±5.17**	**931.34 ±15.22**
**RNAi control**	**760.63 ±6.05**	**846.80 ±5.90**
**p-value**	0.0013*	0.0033*
**Comparison of WAMW of extracted bound sugars in XTH43-OE and XTH43-RNAi roots**		
	**Loosely bound sugars**	**Tightly bound sugars**
**XTH43-OE**	**741.69 ±9.10**	**860.94 ±10.57**
**XTH43-RNAi**	**775.35 ±4.64**	**916.75 ±8.13**
**p-value**	0.0151*	0.0069*
**Comparison of WAMW of extracted bound sugars in RNAi control vs XTH43-OE roots**		
	**Loosely bound sugars**	**Tightly bound sugars**
**RNAi control**	**760.63 ±6.05**	**846.80 ±5.90**
**XTH43-OE**	**741.69 ±9.10**	**860.94 ±10.57**
**p-value**	**0.0792**	**0.154**
**Comparison of WAMW of extracted bound sugars in OE control vs XTH43-RNAi roots**		
	**Loosely bound sugars**	**Tightly bound sugars**
**OE control**	**813.86 ±5.17**	**931.34 ±15.22**
**XTH43-RNAi**	**775.35 ±4.64**	**916.75 ±8.13**
**p-value**	0.0026*	**0.2228**

*statistically significant, p < 0.05.

The second set of analyses show the loosely-bound sugars have a statistically significant difference of 4.34% in XTH43-OE compared to XTH43-RNAi (Table 3). The tightly-bound sugars have a statistically significant difference of 6.09% in XTH43-OE compared to XTH43-RNAi (Table 3). The results show that while the total amount of sugar and XyG are the same, the lengths of those chains are different.

### Direct counterpart analyses

In analyses presented here, the study focuses in on understanding whether the roots that have been genetically engineered to undergo XTH43-OE or RNAi are becoming more like their counterpart control root. The counterpart is defined as the genotype that the transgenic event is generated to emulate such that XTH43-OE in *G*. *max*_[Williams 82/PI 518671]_ is being engineered to become more like the RNAi control in *G*. *max*_[Peking/PI 548402]_. In contrast, XTH43-RNAi in *G*. *max*_[Peking/PI 548402]_ leads to an outcome where the CWs of those transgenic roots become more like the OE control in *G*. *max*_[Williams 82/PI 518671]_.

**Fig 11:**
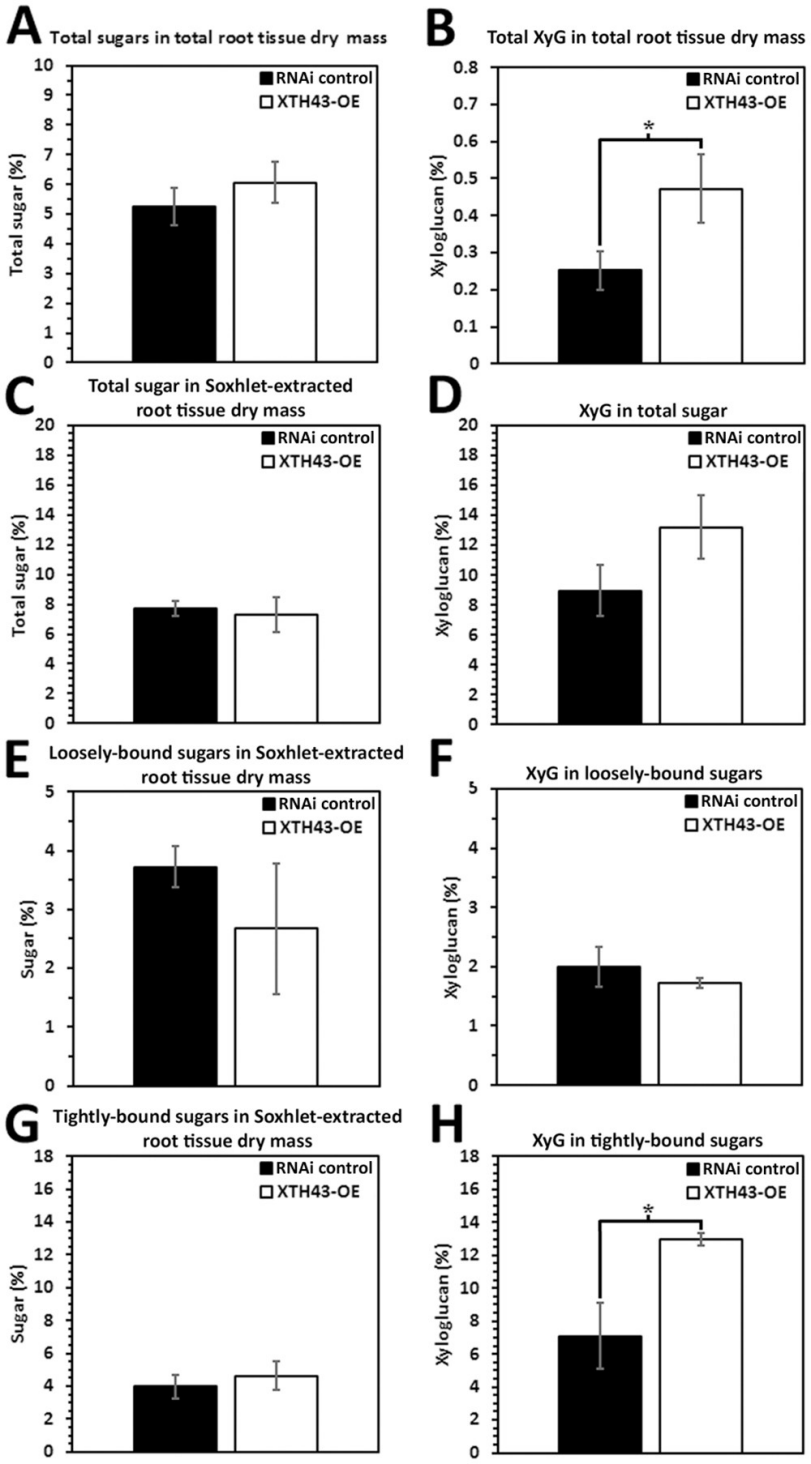
Direct counterpart comparisons made between the RNAi control and XTH43-OE. **A**. Total sugars in total root tissue dry mass (p = 0.3592). **B**. Total XyG in total root tissue dry mass (p = 0.0427). **C**. Total sugar in Soxhlet-extracted root tissue dry mass (p = 0.7756). **D**. Total XyG in total sugar (p = 0.0544). **E**. Loosely-bound sugar in Soxhlet-extracted root tissue dry mass (p = 0.1917). **F**. XyG in loosely-bound sugar (p = 0.2149). **G**. Tightly-bound sugar in Soxhlet-extracted root tissue dry mass (p = 0.0764). **H**. XyG in tightly-bound sugar (p = 0.2070). (*) represents statistically significant difference p ≤ 0.05 calculated by t-test using Welch Two Sample t-test. The results are not statistically significant between the two genotypes. Error bars represent standard error. OE experiments are performed in the *H*. *glycines*-susceptible *G*. *max*_[Williams 82/PI 518671]_. RNAi experiments are performed in the *H*. *glycines*-resistant *G*. *max*_[Peking/PI 548402]_.

Counterpart comparisons of the RNAi control to XTH43-OE do not identify any statistically significant differences in relative amounts of total sugars in total root dry mass, Soxhlet-extracted root dry mass, loosely-bound or tightly-bound sugars (Fig 11A, 11C, 11E and 11G, **respectively**). For XyG in the whole tissue dry mass the RNAi control has a statistically significant 1.88-fold difference compared to XTH43-OE (Fig 11B). To determine where that difference is coming from, when examining the tightly-bound sugars, the RNAi control is shown to have a statistically significant 1.76 fold increase in XyG (Fig 11H). No statistically significant differences occur in XyG relative amounts in Soxhlet-extracted root tissue or loosely-bound sugars (Fig 11C, 11D, 11E and 11F, **respectively**). The amount of XyG found in the Soxhlet-extracted root sample when comparing RNAi control to XTH43-OE is just under the threshold to be found statistically different (Fig 11D). However analyses that employ the OE control as compared to XTH43-OE found a statistically significant difference just over the accepted threshold (p ≤0.05) (Fig 3B). Consequently, it appears as though the statistically significant changes in CW sugars are coming from tightly-bound XyG.

**Fig 12:**
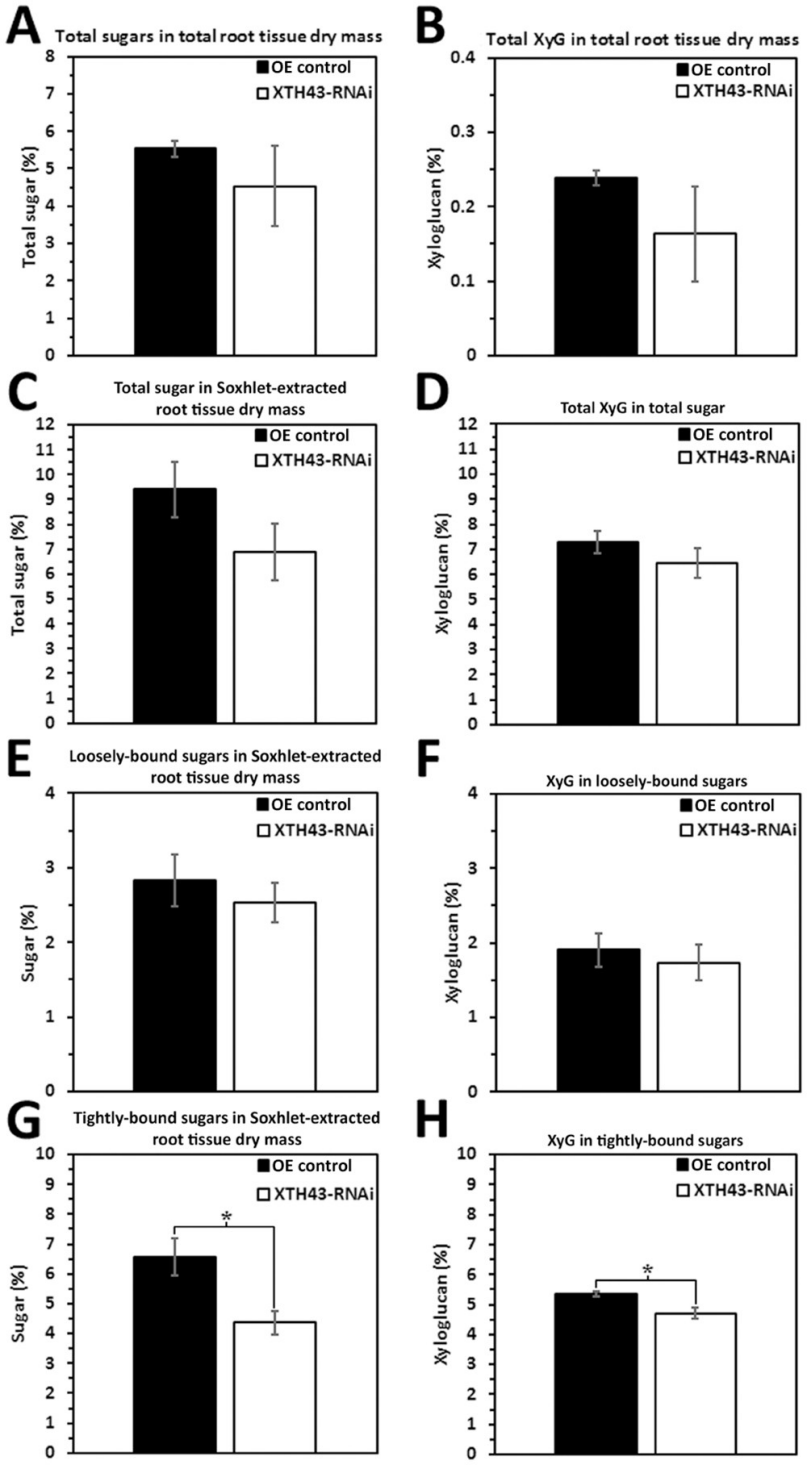
Direct counterpart comparisons made between the OE control and XTH43-RNAi. **A**. Total sugars in total root tissue dry mass (p = 0.4103). **B**. Total XyG in total root tissue dry mass (p = 0.1140). **C**. Total sugar in Soxhlet-extracted root tissue dry mass (p = 0.0520). **D**. Total XyG in total sugar (p = 0.1214). **E**. Loosely-bound sugar in Soxhlet-extracted root tissue dry mass (p = 0.5333). **F**. XyG in loosely-bound sugar (p = 0.6210). **G**. Tightly-bound sugar in Soxhlet-extracted root tissue dry mass (p = 0.0417). **H**. XyG in tightly-bound sugar (p = 0.0300). (*) represents statistically significant difference p ≤ 0.05 calculated by t-test using Welch Two Sample t-test. The results are not statistically significant between the two genotypes. Error bars represent standard error. OE experiments are performed in the *H*. *glycines*-susceptible *G*. *max*_[Williams 82/PI 518671]_. RNAi experiments are performed in the *H*. *glycines*-resistant *G*. *max*_[Peking/PI 548402]_.

Counterpart analyses of the OE control and the XTH43-RNAi show no statistically significant differences in total sugars or XyG relative amounts in total root dry mass, Soxhlet-extracted root dry mass, or loosely-bound sugars (Fig 12A, 12B, 12C, 12D, 12E and 12F). However, a statistically significant difference exists in the tightly-bound sugars amounts of Soxhlet-extracted root tissue and XyG relative amount in tightly-bound sugars. The tightly-bound sugars in the OE control are 1.51-fold different (Fig 12G). Notably, counterpart comparison of the total XyG found in the total dry mass of OE control in relation to XTH43-RNAi (Fig 12B) show no statistically significant differences while the earlier comparison of RNAi control to XTH43-RNAi show a difference (Fig 4B). This result could be attributed to the large standard error found in the XTH43-RNAi sample. Furthermore, the comparison of the XyG in total sugars occurring between the RNAi control and XTH43-RNAi samples (Fig 12D) are statistically the same while the earlier comparison of RNAi control to the XTH43-RNAi samples are statistically significant, but just over that significance threshold (Fig 4D). Lastly, the comparison between the OE control and XTH43-RNAi samples (Fig 12E) show no statistically significant difference while, in contrast, the earlier RNAi control comparison to XTH43-RNAi (Fig 4E) are statistically different. The results indicate that the XTH43-RNAi decreases the relative amounts of tightly-bound sugars to an even greater extent that what is normally found in *G*. *max*_[Williams 82/PI 518671]_ and that at least some of this loss comes from a decrease in the amount of XyG.

### Direct counterpart GPC analyses of loosely-bound sugars

**Fig 13:**
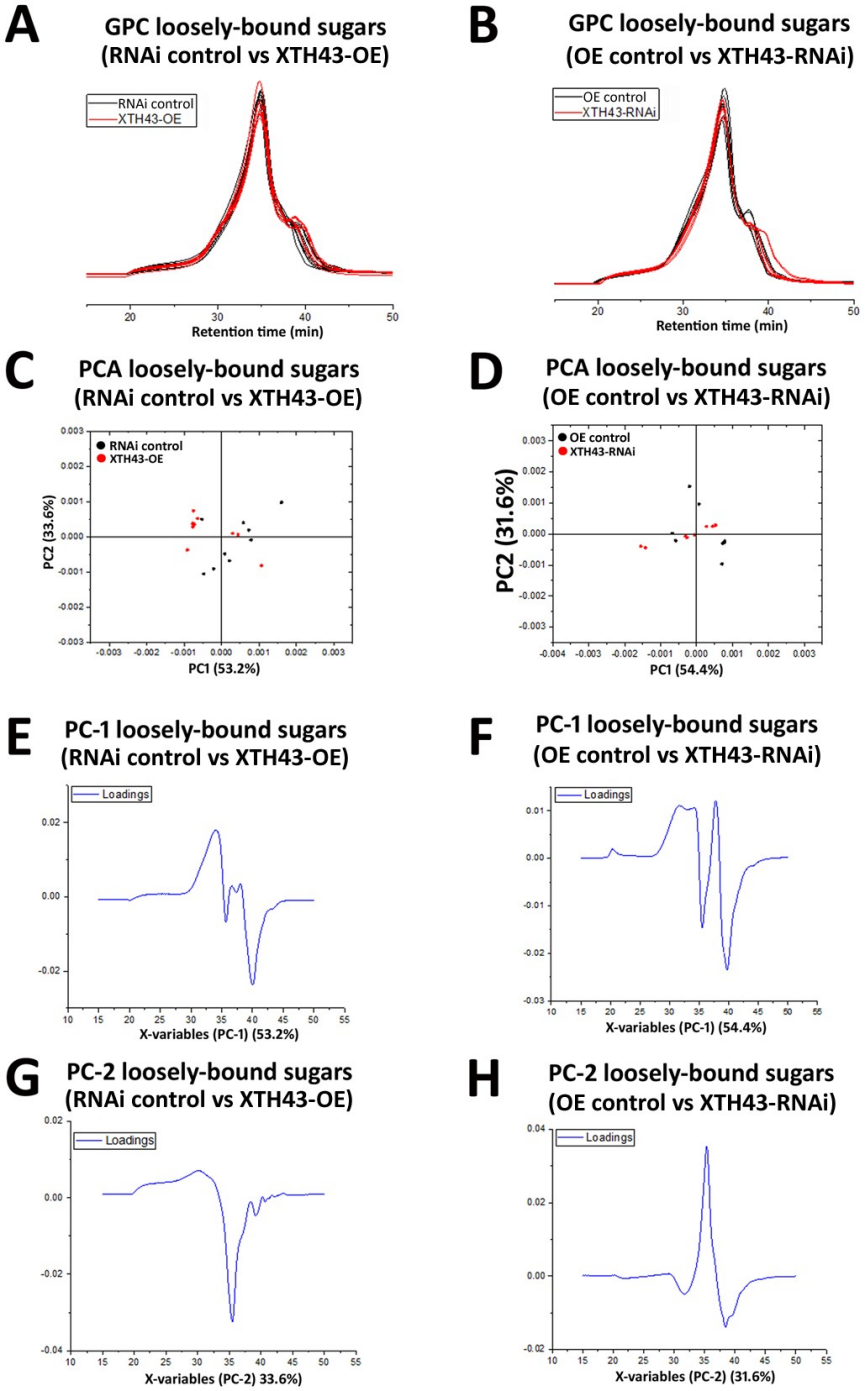
Direct counterpart GPC analysis of loosely-bound sugars in OE control and XTH43-RNAi samples and RNAi control and XTH43-RNAi samples. **A**. Area normalized chromatogram of loosely-bound sugars in OE control (black) and XTH43-RNAi (red) samples. **B**. Area normalized chromatogram of loosely-bound sugars in RNAi control (black) and XTH43-OE (red) samples. **C**. PC-1/PC-2 score plot of OE control (black) and XTH43-RNAi sample spectra of loosely-bound sugars. **D**. PC-1/PC-2 score plot of RNAi control (black) and XTH43-OE sample spectra of loosely-bound sugars. **E**. PC-1 (OE control) loading. **F**. PC-1 (RNAi control) loading. **G**. PC-2 (XTH43-RNAi) loading. **H**. PC-2 (XTH43-OE) loading. OE experiments are performed in the *H*. *glycines*-susceptible *G*. *max*_[Williams 82/PI 518671]_. RNAi experiments are performed in the *H*. *glycines*-resistant *G*. *max*_[Peking/PI 548402]_.

GPC and PCA analyses further examine the CW composition of the OE control and the RNAi control in relation to their counterpart XTH43-RNAi and XTH43-OE roots, respectively. No differences occur in loosely-bound sugars spectra in the OE control as compared to XTH43-RNAi (Fig 13). Loosely-bound sugars of RNAi control and XTH43-OE also show no difference in GPC spectra (Fig 13). PCA analyses confirm no significant differences occurring between the compared sample groups. This result is expected since both the OE control and XTH43-RNAi roots are *H*. *glycines*-susceptible.

### Direct counterpart GPC analyses of tightly-bound sugars

**Fig 14:**
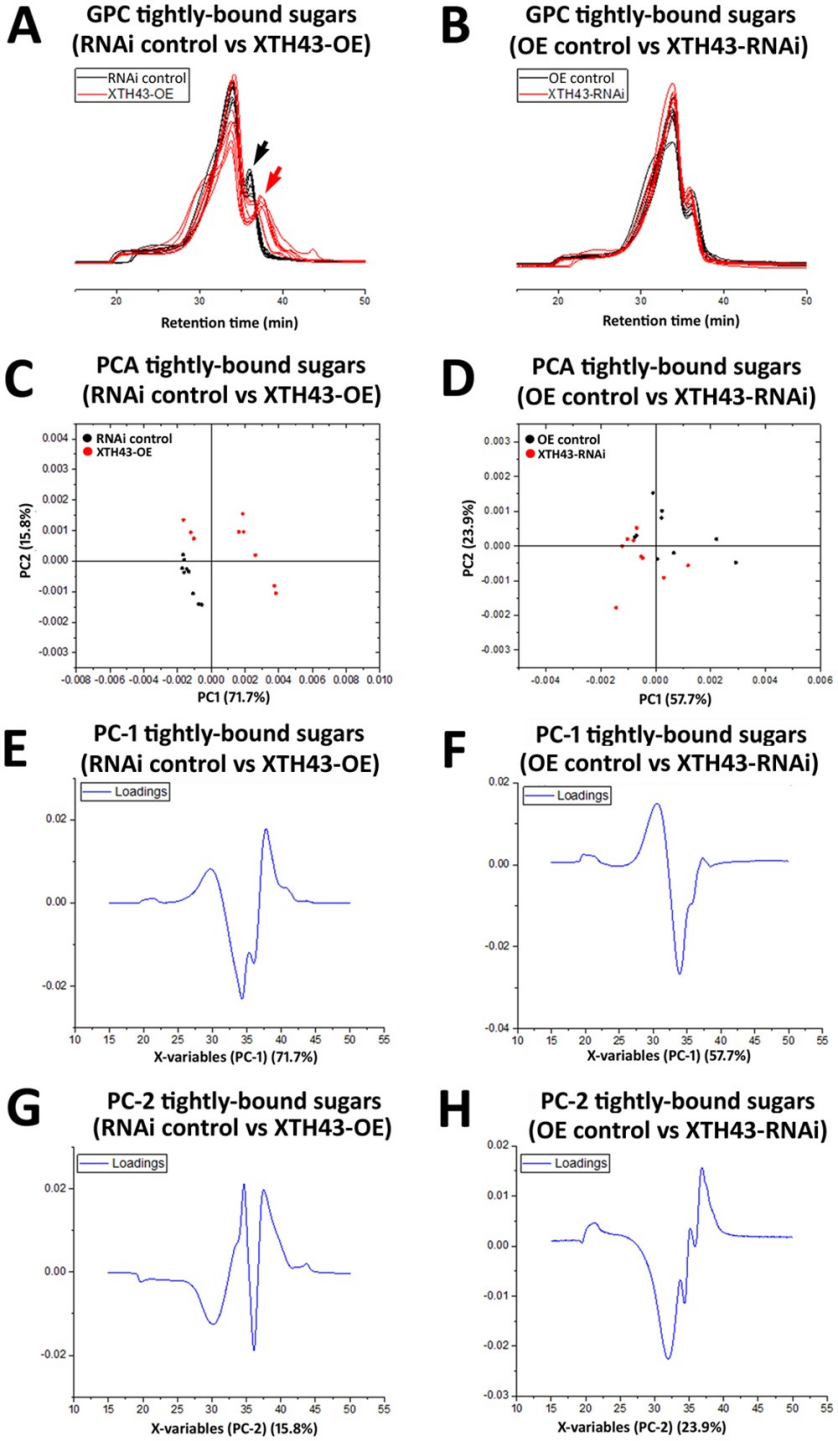
Direct counterpart GPC analysis of tightly-bound sugars in OE control and XTH43-RNAi samples and RNAi control and XTH43-RNAi samples. **A**. Area normalized chromatogram of tightly-bound sugars in OE control (black) and XTH43-RNAi (red) samples. **B**. Area normalized chromatogram of tightly-bound sugars in RNAi control (black) and XTH43-OE (red) samples. **C**. PC-1/PC-2 score plot of OE control (black) and XTH43-RNAi sample spectra of tightly-bound sugars. **D**. PC-1/PC-2 score plot of RNAi control (black) and XTH43-OE sample spectra of tightly-bound sugars. **E**. PC-1 (OE control) loading. **F**. PC-1 (RNAi control) loading. **G**. PC-2 (XTH43-RNAi) loading. **H**. PC-2 (XTH43-OE) loading. OE experiments are performed in the *H*. *glycines*-susceptible *G*. *max*_[Williams 82/PI 518671]_. RNAi experiments are performed in the *H*. *glycines*-resistant *G*. *max*_[Peking/PI 548402]_.

In examining tightly-bound sugars, GPA and PCA counterpart analyses identify a spectral shift in the RNAi control as compared to the XTH43-OE samples (Fig 14). The result confirms the CW of *G*. *max*_[Williams 82/PI 518671]_ chains are becoming more like the RNAi control in *G*. *max*_[Peking/PI 548402]_. However, the sugar chains are becoming even shorter than *G*. *max*_[Peking/PI 548402]_, possessing a spectral shift even beyond what already exists in the resistant *G*. *max*_[Peking/PI 548402]_ genotype. In contrast, no difference is occurring between the OE control and the XTH43-RNAi tightly-bound sugars in GPC and PCA analyses (Fig 14). The result confirms that the CW chains of the XTH43-RNAi transgenic roots are becoming more like the OE control roots in the susceptible *G*. *max*_[Williams 82/PI 518671]_ genotype.

### Direct counterpart WAMW analyses

WAMW analyses identify no statistically significant difference in loosely-bound sugars of the RNAi control compared to XTH43-OE (Table 3). Likewise, as expected, comparisons of the tightly-bound sugars of the RNAi control to the XTH43-OE plants identify no statistically significant difference (Table 3). While many changes in tightly-bound sugars occur throughout this study, they are statistically the same here. This result supports the hypothesis that the XTH43-OE in *G*. *max*_[Williams 82/PI 518671]_ leads to changes in sugar composition that makes its CW more like the CW of *G*. *max*_[Peking/PI 548402]_. In contrast, the results also support the hypothesis that XTH43-RNAi in *G*. *max*_[Peking/PI 548402]_ leads to changes in sugar composition that makes its CW more like *G*. *max*_[Williams 82/PI 518671]_.

Comparisons of the loosely-bound sugars identify a statistically significant lower level of -4.73% in the XTH43-RNAi roots as compared to the OE control (Table 3). The loosely-bound sugars in the XTH43-RNAi roots, therefore, are not as long or longer that the OE control roots. Therefore, the RNAi process is not making the length of these loosely-bound sugars like what is found in the OE control in the susceptible *G*. *max*_[Williams 82/PI 518671]_ genotype. However, as expected, comparisons of the tightly-bound sugars of the OE control to the XTH43-RNAi roots result in no statistically significant difference (Table 3). This result means that the tightly-bound sugars (OE control) is statistically the same as the XTH43-RNAi roots.

## Discussion

**Fig 15:**
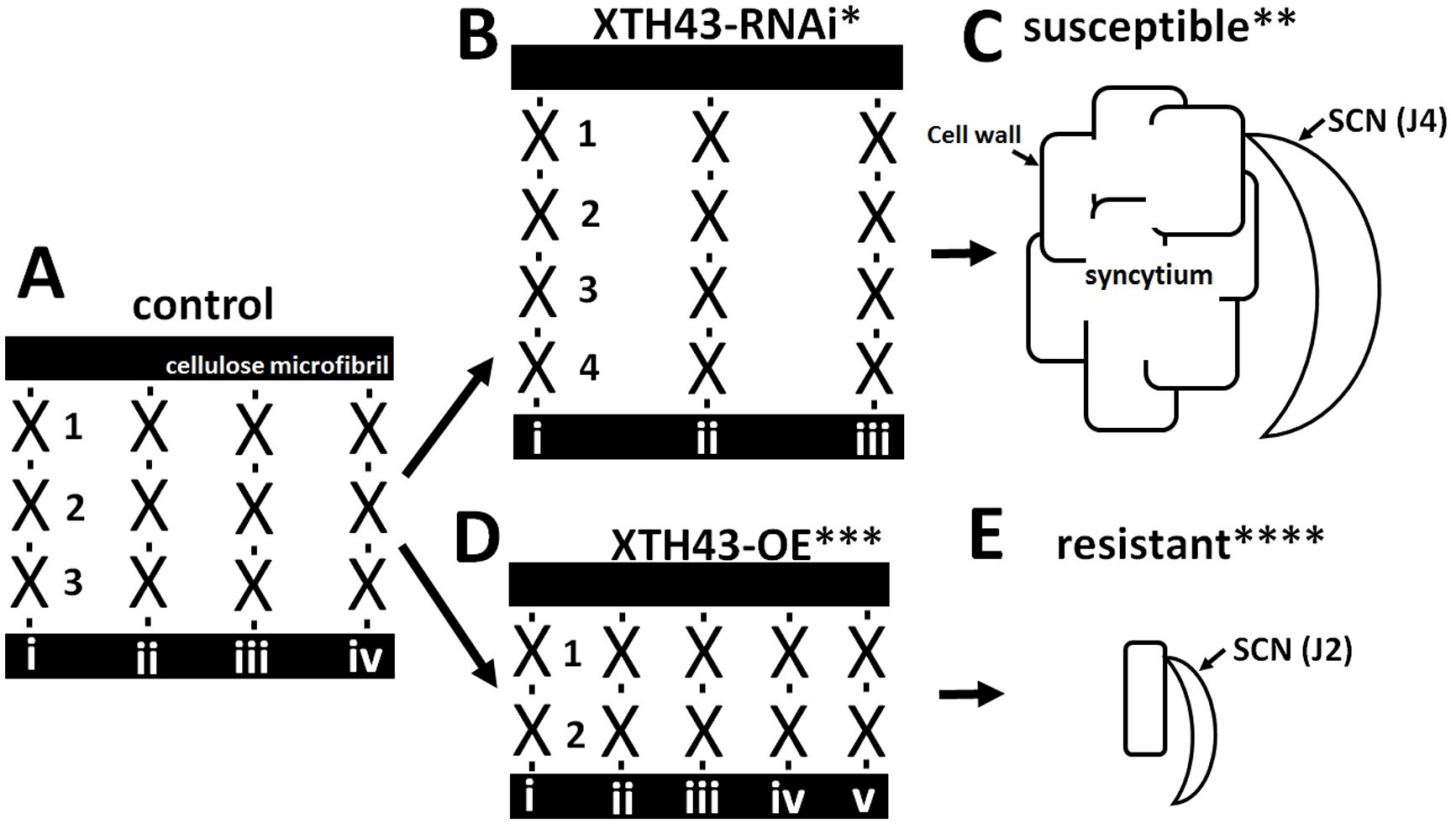
Model showing cellulose microfibrils with bound XyG chains presented only for diagrammatic purposes. **A**. 3 linked XyG monomers (1–3) with 4 chains (i-iv). The links occurring between the XyG monomers are shown as a black dash. **B**. In XTH43-RNAi expressing roots in the *H*. *glycines*-resistant *G*. *max*_[Peking/PI 548402]_ there is an increase in the number of XyG in each chain (1–4), but fewer XyG chains (i-iii). **C**. Cartoon of an XTH43-RNAi root in the *H*. *glycines*-resistant *G*. *max*_[Peking/PI 548402]_ showing *H*. *glycines* developing a syncytium. **D**. In XTH43-OE expressing roots in the *H*. *glycines*-susceptible genotype *G*. *max*_[Williams 82/PI 518671]_ there is an decrease in the number of XyG in each chain (1–2), but more chains (i-v). **E**. Cartoon of an XTH43-OE root *H*. *glycines*-susceptible genotype *G*. *max*_[Williams 82/PI 518671]_ showing *H*. *glycines* where the syncytium failed to expand and form. X, XyG monomer. * XTH43-RNAi showing an increase in XyG chain length. ** the XTH43-RNAi results in an *H*. *glycines*-susceptible outcome as shown in Fig 2B as compared to the OE control in Fig 2A. *** XTH43-OE showing an decrease in XyG chain length and more of those chains. ****The XTH43-OE results in an *H*. *glycines*-resistant outcome as shown in Fig 2D as compared to the RNAi control in Fig 2C.

The results presented here supports XTH43 increasing XyG content, but specifically tightly-bound XyG so that XyG chain length is decreased in mass while increasing the number of shorter XyG chains. Consequently, a cell wall environment may be produced that interferes with its ability to expand into a syncytium, either leading to a resistant reaction or acting along with other processes that result in a resistant reaction (Fig 15). The results reflect the importance of the plant secretion system functioning in defense in the *G*. *max*-*H*. *glycines* pathosystem and that XTH43, as a secreted protein, functions in the process [47,48,59]. Furthermore, XTH43 transcript abundance increases as a consequence of the overexpression of components of both effector triggered immunity (ETI) and pathogen activated molecular pattern triggered immunity (PTI), as well as treatment with the bacterial effector harpin [48,50,62,73]. This analyses attempts to understand XTH43 function.

### Suppressed XTH43 expression increases *H*. *glycines* parasitism

XTH43-RNAi, expectedly, facilitates *H*. *glycines*’ ability to parasitize *G*. *max*. These results compliment prior overexpression experiments that show XTH43 overexpression interferes with the ability of *H*. *glycines* females to mature into cysts [48]. The currently understood roles of XTH lend to the idea presented here through experimental data that the XTH43 protein appears to have a role in re-arranging or re-modelling of XyG to combat *H*. *glycines* parasitism. The experiments presented here show XTH43-RNAi leads to a statistically significant decrease in root mass. These results are in contrast to prior overexpression results that showed no effect [48]. Both CW composition analyses and GPC have been performed to determine the components of the CW and understand the nature of XyG as it relates to experimentally-driven modulations in XTH43 expression. It is noted that other direct or indirect processes that relate to defense may also associate with the role(s) that XTH43 has. Furthermore, potential non-specific effects caused by RNAi may contribute to the altered XyG content observed in the experiments presented here. However, in obtaining the opposite effect in the XTH43-OE experiments leads us to conclude the results have a level of specificity limited to RNAi of XTH43. Furthermore, only a specific type of XyG, the tightly-bound fraction, is affected.

### Measurement of total sugars in XTH43-overexpressed and RNAi plants

No difference in the amount (in percent) of total sugars and XyG in Soxhlet-extracted total sugars from *G*. *max* root total root tissue dry mass occur in the XTH43-OE or XTH43-RNAi roots, compared to their respective controls. However, an increase in XyG occurs in Soxhlet-extracted total sugars from *G*. *max* XTH43-OE root dry mass with a concomitant decrease in XyG in the XTH43-RNAi roots as compared to the respective control. Further analyses of the XTH43-OE and XTH43-RNAi roots do not identify any statistically significant difference in total sugars or XyG as compared to the respective control. Consequently, the difference in XyG is limited to a specific type of XyG. A statistically significant difference occurs in the amount (in percent) of XyG in Soxhlet-extracted total sugars (4% +24% KOH). However, the type of XyG had been unclear. Nishikubo *et al*. (2011) [49] reported an increase in tightly-bound XyG in the XTH-OE roots as compared to wild type *Populus* sp. by approximately 20%, while decreasing the loosely-bound fraction. The analyses of loosely-bound total sugars identify no statistically significant differences between XTH43-OE and the OEcontrol. This observation differs from Nishikubo *et al*. (2011) [49]. Many different XTHs exist in plant genomes, likely performing specialized functions that complicate comparison. The analysis presented here only identified the amount (in percent) of XyG in Soxhlet-extracted loosely-bound sugars from *G*. *max* roots. GPC and PCA analyses show that while the amount of sugar is the same, there exists a decrease in the length of those chains in the XTH43-OE roots compared to the OE control. Consequently, the analyses allow for the distinction between the amount of sugars from the length of those polysaccharide chains. In contrast, a statistically significant decrease in loosely-bound sugars occurs in the XTH43-RNAi roots compared to its RNAi control. However, in the XTH43-RNAi roots GPC and PCA analyses demonstrate there is no difference in the length of those chains as compared to the RNAi control. The loosely-bound sugars may be recruited in different ways for other purposes. However, they do not appear to be XyG because comparative analyses of loosely-bound XyG between XTH43-OE and OE control do not identify any statistically significant differences. An XTH mutant in *A*. *thaliana* results in significantly reduced hemicellulose and total XyG content [74]. This result can be explained by how XyG functions, acting as sites of attachments for other sugars. Consequently, if less tightly-bound XyG exists then there would be fewer binding sites for loosely-bound sugars. An analysis of tightly-bound XyG would make that determination.

Analyses of tightly-bound sugars in the XTH43-OE and XTH43-RNAi roots identify no statistically significant differences as compared to controls. In contrast, a statistically significant increase in XyG occurs in the XTH43-OE roots as compared to its OE control. Furthermore, GPC and PCA analyses demonstrate that the sugar chains are shorter in the XTH43-OE roots as compared to the OE control. Therefore, the tightly-bound sugars have both increased in quantity and became shorter in length. In contrast, a statistically significant decrease in XyG content occurs in the XTH43-RNAi roots as compared to its RNAi control. However, no difference in the length of those sugar chains occurs in GPC and PCA analyses. These results are consistent with XTH-OE increasing tightly-bound XyG content in *Populus* sp. [49]. It is also consistent with XTH43 becoming expressed specifically after the defense response onset in *G*. *max* to *H*. *glycines* parasitism [47].

The observation of a higher amount of XyG in tightly-bound sugars than loosely-bound sugars suggests that XyG is modified after deposition in the CW. XyG changes soon after deposition, including an increase in size, depolymerization and increment in amount during expansion [75–79]. Several rearrangements do occur during transitional stages of the CW. A transition of loosely-bound sugars to tightly-bound to cellulose microfibrils into a tightly-bound network happens along with these transitions [49,80]. While modified XTH activity has a mixed effect on XyG, a definitive role of XTH occurs in which XTH activity promotes XyG incorporation into the CW of tightly-bound fraction [3,42,49,74,81,82]. Changes in mono-sugar composition of hemicellulose fractions occur. The results demonstrate higher XyG amounts correlate with higher XTH43 expression and lower amounts to be correlated with decreased expression. These observations support the hypothesis that XTH activity incorporates newly synthesized XyG to its network through transglycosylation, increasing the net amount of XyG content of the CW. Consequently, in cells that do not synthesize XyG, XTH may act only in their rearrangement. XTH is considered a key component for maintaining the chain length and extensibility of cellulose-XyG network [83,84]. The function of XTH appears to be more complex than a simple role in cell expansion catalyzed through depolymerization of XyG as reported [3,7,43,75,83,85].

### Weight average molecular weight of XyG chains

XTH43-OE leads to a lower molecular mass than the OE control samples while XTH43-RNAi leads to a higher molecular mass than RNAi control samples. These results indicate that shortening of the XyG chain length in XTH43-OE and increasing chain length in XTH43-RNAi roots occurs as compared to their respective control roots. Nishikubo *et al*. (2011) reported a shift toward lower molecular mass XyG within the main peak and higher content of low molecular mass XyG from *Populus* XTH-OE roots [49]. Similarly, Herbers *et al*. (2001) noted an increase in XyG size by 20% when XTH expression is suppressed [81]. Although, there have been dubious explanations of the role of XTH in CW morphogenesis in many plant species, high expression of XTH in the region of active CW formation indicates a role(s) in CW deposition and remodeling even after enlargement has ceased [31,86–90]. The evidence argues for XTH43 involvement in the incorporation of XyG molecules into the CW through transglycosylation. The observation of lower MW XyG in XTH43-OE roots and higher MW XyG in XTH43-RNAi roots than their respective controls might be associated with CW strengthening by XTH-mediated remodeling of XyG chain in CW architecture. The XyG chain length estimation, based on WAMW obtained through GPC, must be regarded with caution as it is sensitive to conformation [91]. It is plausible that the observation of MW sugars are not all XyG, but also the result of multiple polysaccharide reorganizations occurring in the CW [7,75,92,93]. Thus, the MW data must be best interpreted as relative rather than absolute size. Also, the conformation of XyG may vary on the degree of substitution of side chain residues, acetylation pattern and different biological sources and stages of the same material that could attribute to the differences. High pressure liquid chromatography (HPLC) would provide more extensive data regarding the XyG chain [66].

### Direct comparisons demonstrate the similarities between the CWs of different *G*. *max* genotypes before *H*. *glycines* infection

Analyses have directly compared the CWs of the OE control made in *G*. *max*_[Williams 82/PI 518671]_ and RNAi control made in *G*. *max*_[Peking/PI 548402]_. The measured CW sugars and XyG show no statistically significant differences. This outcome is reflected in the GPC and PCA analyses, consistent with XTH43 expression occurring specifically in syncytia undergoing a defense response. However, WAMW analyses demonstrate that the loosely and tightly-bound sugars are shorter in the RNAicontrol. It is unclear what the discrepancy between the percent sugars and XyG and GPC and PCA analyses are reflecting, but if the loosely and tightly-bound sugars are shorter in the RNAi control in the *H*. *glycines*-resistant *G*. *max*_[Peking/PI 548402]_ then it is possible it has a CW environment in place to potentially hasten the impairment of *H*. *glycines* parasitism.

In contrast, direct comparisons of the XTH43-OE to XTH43-RNAi show differences in XyG in total root tissue dry mass, total sugar and tightly-bound sugar. These results are supported by the WAMW analyses showing shorter tightly-bound sugar chains occurring in the XTH43-OE roots as compared to the XTH43-RNAi roots. No difference in XyG occurs in loosely-bound sugars, reflected in the GPC and PCA analyses. However, the WAMW analyses identify shorter loosely-bound sugar chains occurring in the XTH43-OE roots as compared to the XTH43-RNAi roots. The results indicate that XTH43-OE can increase the amount of XyG while also decrease the length of XyG beyond what is found in an XTH43-RNAi-impaired *H*. *glycines*-resistant *G*. *max*_[Peking/PI 548402]_. The question then became whether XTH43-OE made the CW of *G*. *max*_[Williams 82/PI 518671]_ similar in composition to *G*. *max*_[Peking/PI 548402]_, the purpose of the counterpart analyses.

### Counterpart analyses determine the extent of modification the CWs are undergoing

Counterpart analyses compare the RNAi control to the XTH43-OE roots, showing the total percent sugar composition remains unchanged. This observation is reflected in the GPC, PCA and WAMW analyses for the loosely-bound sugars. In contrast, the percent analyses of XyG in the RNAi control as compared to XTH43-OE demonstrates an increase in XyG in XTH43-OE in analysis of total XyG in root tissue dry mass and tightly-bound sugars. These observations are reflected in the GPC and PCA analyses of tightly-bound sugar analyses. However, no difference occurs for loosely or tightly-bound sugars by WAMW. The results indicate the XTH43-OE, driven by the FMV promotor, is leading to the production of higher amounts of its protein than what would be found in the uninfected *H*. *glycines*-resistant *G*. *max*_[Peking/PI 548402]_, possibly recapitulating levels found during the actual defense response.

Counterpart analyses of OE control to XTH43-RNAi allow for the determination of how similar the CWs become by the impairment of XTH43 expression. The results demonstrate that the tightly-bound sugar and XyG are lower in the XTH43-RNAi roots. However, no difference in the length of those chains occurs by the GPC or PCA analyses. Consequently, the impairment appears to mainly be a decrease in tightly-bound sugars and XyG. However, while GPC and PCA analyses show that no effect on the length of those chains occurs the WAMW analyses measure a decrease in loosely-bound sugars.

### Relating XTH43 function to defense

The XyG backbone attributes a relatively rigid conformation whose flexibility is mainly dependent on the side chain and their interaction to other CW components [94,95]. In performing that task, it can be surmised that altering XyG changes the plant’s pathogen defense ability. XTHs as secreted proteins that undergo glycosylation imply that the Golgi apparatus functions prominently in the modification of XTH43 and the production of XyG that are secreted into the apoplast for CW modification. XTH43 belongs to a large gene family composed of at least 54 members in *G*. *max* [48]. In the first RNA seq analyses of RNA isolated from syncytia undergoing the defense response XTH43 is among the more highly expressed genes [47]. The analyses indicate a specific structural role exists for XTH43, relating to defense. The similarity of sugar and XyG content found between the *H*. *glycines*-susceptible *G*. *max*_[Williams 82/PI 518671]_ and *H*. *glycines* resistant *G*. *max*_[Peking/PI 548402]_, in uninfected roots, demonstrate that XTH43 functions locally at the site of parasitism to support a defense response. XTH43 functions to increase the amount of XyG and shorten the lengths of its polysaccharide chains and make more chains, consistent with the original histological observations of Ross [96]. Based on the XTH43 expression pattern these processes are occurring even though the nematode is delivering effectors into the plant, indicating the defense response is potent and that the Golgi apparatus performs a prominent role.

The Golgi apparatus is a structure composed of a number of stacked cisternae which function in packaging proteins and other materials into membrane-bound vesicles and glycosylation. Residing at the intersection of endocytic, lysosomal and secretory pathways, the Golgi apparatus serves as a site where proteins and other metabolites are produced and/or modified as they are enclosed within vesicles for transport to various destinations within the cell. Consistent with the observations presented here, the import of xyloglucan precursors and location of enzymes that function in the production of xyloglycan occurs at/within the Golgi apparatus [17,97,98]. The *Saccharomyces cerevisiae* syntaxin, suppressors of the erd2-deletion 5 (Sed5p) binds directly to Sec17p, a homolog of the *G*. *max* major *H*. *glycines* resistance gene *rhg1* gene α-SNAP, in processes that transport cargo through the Golgi apparatus [99,100]. Furthermore, Sed5p is involved in retrograde trafficking through its interaction with the conserved oligomeric Golgi (COG) complex, a process involving the Golgi apparatus [101]. The COG complex is composed of 8 proteins that function in plant defense, including the *G*. *max* defense response to *H*. *glycines* [102,103]. These observations compliment other work showing the exocyst, also involved in secretion of proteins to the apoplast, functions in the *G*. *max* defense response to *H*. *glycines* parasitism and is consistent with the role that the soluble N-ethylmaleimide-sensitive fusion protein attachment protein receptor (SNARE) has in this process [48,59,103–105]. XTHs have a signal peptide which permits ER targeting and are *N*-glycosylated which would indicate secretory pathway processing, features also predicted for Gm-XTH43 [38–43,48]. XTHs have also been shown to associate with vesicles, an association indicative of regulated trafficking to its site of activity [39,46]. Therefore, the Golgi apparatus likely is involved in processes involving XTH43 during cell wall metabolic processes relating to the defense process.

## Supplemental data

Supplemental files include S1 Table, Primers used in the analysis and S1 Fig, Area normalized GPC chromatograms of dextran standards (25–1,400 kDa).

## Supporting information

S1 Fig Area normalized GPC chromatograms of dextran standards (25–1,400 kD). **A**. 25 kD, 20–120 min; **B**. same as A with 25 kD, 20–40 min region highlighted. **C**. 50 kD, 0–120 min; **D**. same as C with 50 kD, 20–40 min region highlighted. **E**. 80 kD, 0–120 min; **F**. same as E with 80 kD, 0–40 min region highlighted. **G**. 150 kD, 0–120 min; **H**. same as G with 150 kD, 20–40 min region highlighted. **I**. 270 kD, 0–120 min; **J**. same as I with 270 kD, 20–40 min region highlighted. **K**. 670 kD, 0–120 min; **L**. same as K with 670 kD, 20–40 min region highlighted. **M**. 1,400 kD, 0–120 min; **N**. same as M with 1,400 kD, 20–40 min region highlighted. The results show 3 independently-run, independent biological replicates.
(TIF)

S1 Table Primers used in the analysis. (TIF)

## Abbreviations

CW: nematode, cell wall
GPC: gel permeation chromatography
PCA: principal component analyses
WAMW: weight average molecular weight
XTH: Xyloglucan endotransglycosylase/hydrolase
XyG: xyloglucan
