# Supplementary figures and images for: Xyloglucan endotransglycosylase/hydrolase increases tightly-bound xyloglucan and chain number but decreases chain length contributing to the defense response that Glycine max has to Heterodera glycines
Source: PLoS One. 2021 Jan 14;16(1):e0244305. doi: 10.1371/journal.pone.0244305 (PMC7808671; doi:10.1371/journal.pone.0244305)

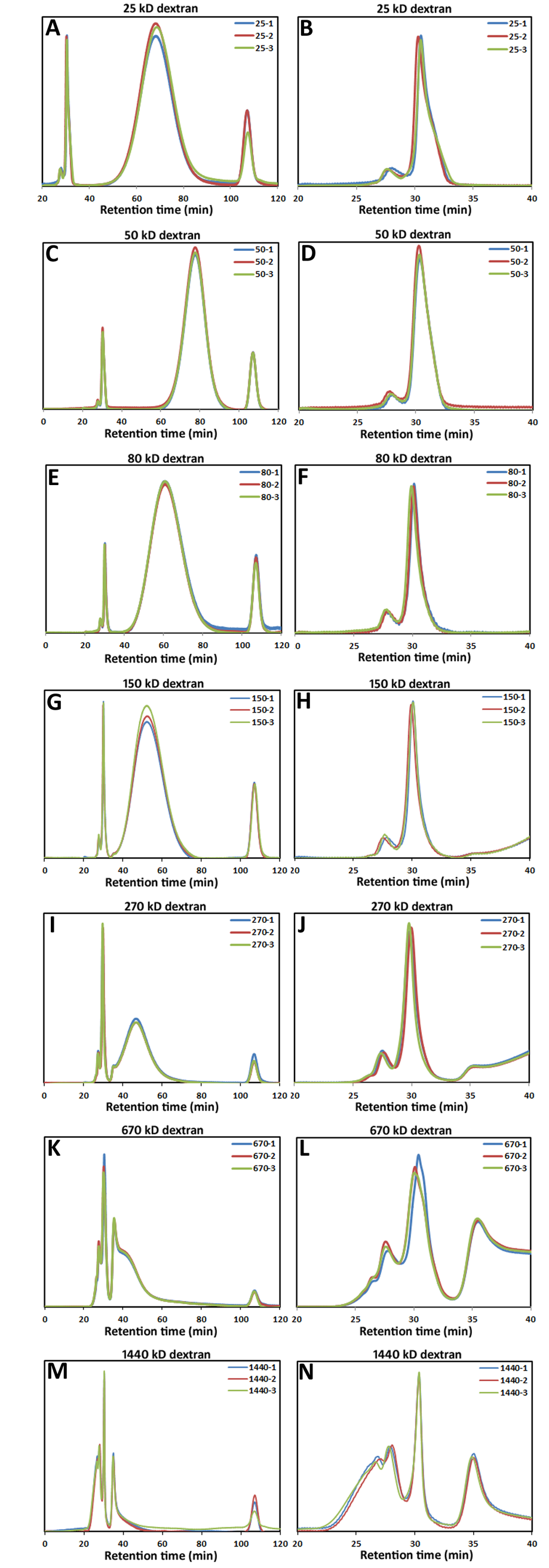

Supplement: S1 Fig — A. 25 kD, 20–120 min; B. same as A with 25 kD, 20–40 min region highlighted. C. 50 kD, 0–120 min; D. same as C with 50 kD, 20–40 min region highlighted. E. 80 kD, 0–120 min; F. same as E with 80 kD, 0–40 min region highlighted. G. 150 kD, 0–120 min; H. same as G with 150 kD, 20–40 min region highlighted. I. 270 kD, 0–120 min; J. same as I with 270 kD, 20–40 min region highlighted. K. 670 kD, 0–120 min; L. same as K with 670 kD, 20–40 min region highlighted. M. 1,400 kD, 0–120 min; N. same as M with 1,400 kD, 20–40 min region highlighted. The results show 3 independently-run, independent biological replicates. (TIF) [file pone.0244305.s001.tif]

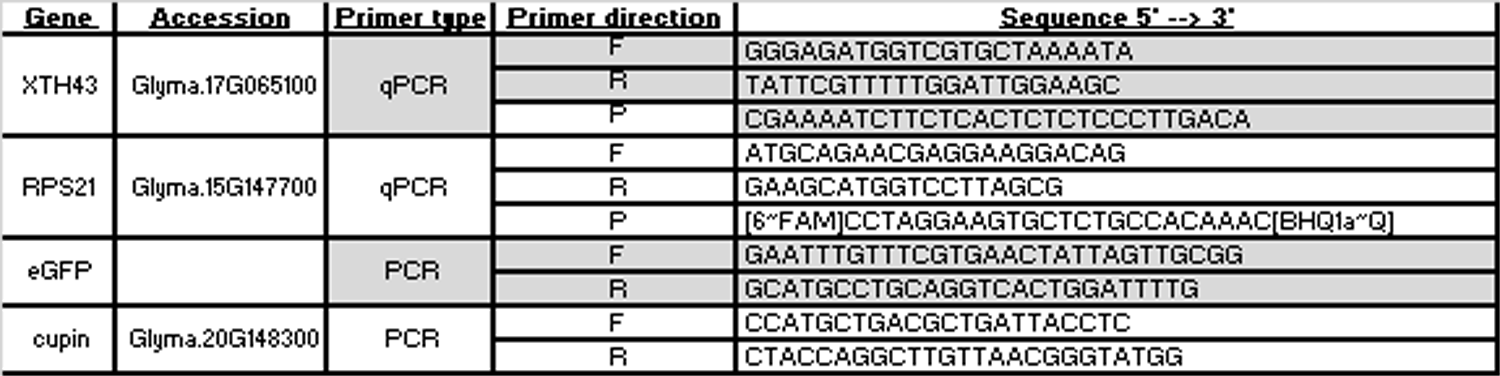

Supplement: S1 Table — (TIF) [file pone.0244305.s002.tif]
